# Circulating extracellular vesicles from early-stage lung cancer patients trigger endothelial activation to drive pre-metastatic niche formation through synergistic miR-29a and C4A signaling

**DOI:** 10.1186/s13046-026-03732-4

**Published:** 2026-05-13

**Authors:** Francesca Pontis, Patrizia Ghidotti, Nicole Ferrario, Camilla Locatelli, Mattia Boeri, Marco Gentili, Miriam Segale, Massimo Moro, Arianna Di Bernardo, Giulia Bertolini, Paola Suatoni, Michele Ferrari, Ugo Pastorino, Loris De Cecco, Marica Ficorilli, Marta Lucchetta, Silvia Brich, Luca Agnelli, Fabio Maiullari, Roberto Rizzi, Claudia Bearzi, Paola Portararo, Sabina Sangaletti, Rossella Crescitelli, Luca Roz, Gabriella Sozzi, Orazio Fortunato

**Affiliations:** 1 Unit of Epigenomics and Biomarkers of Solid Tumors, Fondazione IRCCS Istituto Nazionale Dei Tumori, Milan, Italy; 2 Thoracic Surgery Unit, Fondazione IRCCS Istituto Nazionale Dei Tumori, Milan, Italy; 3 Unit of Integrated Biology of Rare Tumors, Fondazione IRCCS Istituto Nazionale Dei Tumori, Milan, Italy; 4 Pathology Unit 2, Department of Diagnostic Innovation, Fondazione IRCCS Istituto Nazionale Dei Tumori, Milan, Italy; 5 Institute for Biomedical Technologies, National Research Council, Via Fratelli Cervi, 93, 20054 Segrate, Milan Italy; 6 Institute of Physical Chemistry - Polish Academy of Sciences, Warsaw, Poland; 7 Department of Medical-Surgical Sciences and Biotechnologies, Sapienza University of Rome, C.So Della Repubblica 79, 04100 Latina, Italy; 8 Molecular Immunology Unit, Fondazione IRCCS Istituto Nazionale Dei Tumori, Milan, Italy; 9 Sahlgrenska Center for Cancer Research, Department of Surgery, Institute of Clinical Sciences, Sahlgrenska Academy, University of Gothenburg, Gothenburg, Sweden

## Abstract

**Background:**

Metastatic recurrence represents the major clinical challenge in early-stage lung cancer after curative surgery. Here, we investigated the role of circulating extracellular vesicles and particles (EVPs) in promoting formation of pre-metastatic niches (PMNs).

**Methods:**

Plasma-derived EVPs were obtained by ultracentrifugation from pre-surgery blood samples of patients with poor prognosis. Heavy-smokers cancer free individuals were used as control. EVP were characterized following MISEV guidelines. Functional experiments were carried out in vitro in 2D and 3D-bioprinted models as well as in vivo*.*

**Results:**

EVPs from patients with early relapse show distinct molecular profiles, characterized by elevated levels of miR-29a and complement protein C4a. These EVPs preferentially target endothelial cells inducing a pro-inflammatory condition with upregulation of VCAM1 and CXCL1. In turn, endothelial modulation stimulated fibroblast activation and promoted neutrophils recruitment supporting PMNs formation. Mechanistically, we demonstrate that miR-29a and C4A act synergistically through SPARC down-modulation promoting cancer cell colonization. Preconditioning of mouse lungs using EVPs from patients with poor prognosis increased metastatic growth of human tumor cells, which was inhibited by miR-29a blockade.

**Conclusions:**

Circulating EVPs could be novel prognostic biomarkers and key players in PMN formation offering new targets to reduce relapses in lung cancer.

**Graphical Abstract:**

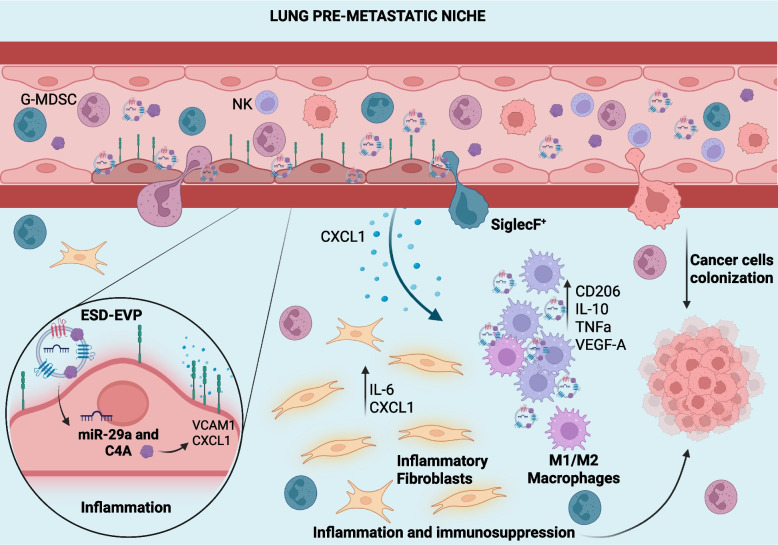

**Supplementary Information:**

The online version contains supplementary material available at 10.1186/s13046-026-03732-4.

## Background

Due to its high incidence, aggressive nature and limited treatment options, lung cancer (LC) is the leading cause of cancer-related deaths [1]. Most LC-associated deaths are caused by advanced or metastatic disease. For instance, the 5-year survival rate of patients with metastatic LC is significantly lower than that of patients diagnosed with localized cancer (5% vs. 57%) [1]. Almost half of early stage patients develop incurable metastases within 2 years of diagnosis, despite complete resection of the primary localized tumor [2].

It is now well established that primary tumors can alter distant organs to create a microenvironment conducive to cancer cell seeding called premetastatic niche (PMN). PMN formation is a finely tuned process that leads to the alteration of several microenvironment components, including vascular leakiness, remodeling of the extracellular matrix and modulation of host immunity [3].

PMN establishment requires systemic activation and the cooperation of several soluble factors, including cytokines, growth factors and extracellular vesicles and particles (EVPs), both secreted by cancer or stromal cells in the tumor (micro)environment [4, 5].

EVPs have recently emerged as key mediators of long-distance biological communications. As membrane-enclosed vesicles, EVPs can provide stability and protection to their cargo and efficiently deliver bioactive molecules (proteins, nucleic acids, lipids and metabolites) such to reach the target tissue where PMN will form [6]. EVPs released from primary tumors have been implicated in several aspects of PMN formation, including immune modulation, angiogenesis and extracellular matrix remodeling [5, 7, 8]. Circulating EVPs represent an easily accessible source of biomarkers [9, 10].

Owing to the rapid spread of LC, only a few studies have been conducted to understand the initial phases of LC metastasis and little is known about the role of circulating EVPs in lung PMN formation. A thorough investigation of the role of circulating-EVPs in PMN formation in lung cancer is crucial for identifying patients at high risk of recurrence and metastasis.

The purpose of this study was to examine plasma-EVPs with the goal of identifying novel prognostic biomarkers and to investigate their potential functional role in early-stage lung cancer patients in the development of metastasis.

## Results

### EVPs from early-stage lung cancer patients with poor-prognosis display distinctive molecular signatures in both surface markers and cargo composition

**Fig. 1:**
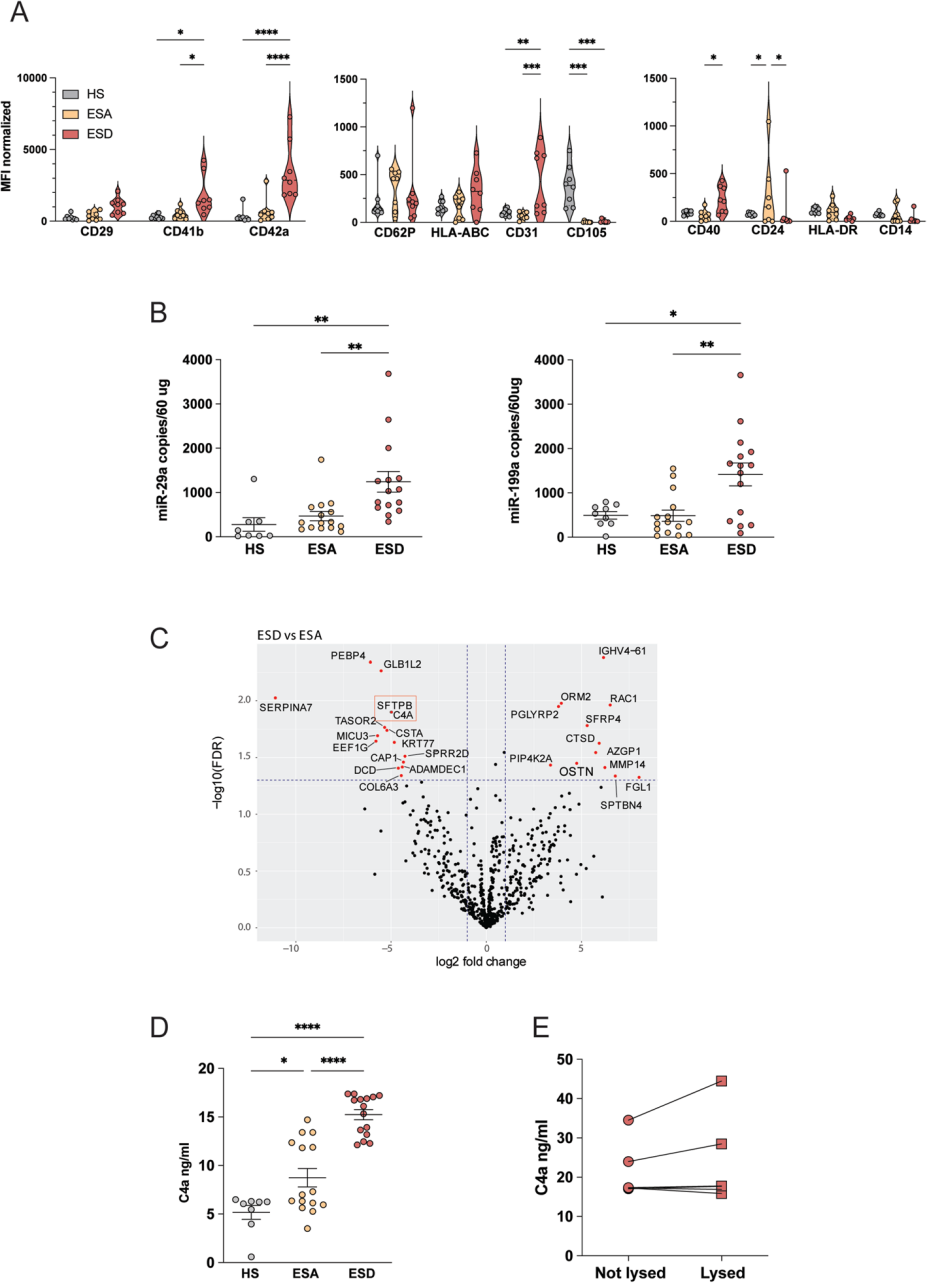
Characterization of plasma derived EVPs from early-stage lung cancer patients and heavy-smokers individuals. **A** Profiles of plasma-derived EVPs surface markers determined by MACSplex kit. The values are expressed as median fluorescence intensity normalized on tetraspanins expression (*n* = 8 per group). **B** Absolute quantification (copies/µl) of miR-29a and miR-199a content in EVPs isolated from HS (*n* = 8), ESA (*n* = 15) and ESD (*n* = 15) patients. The data are expressed as mean ± S.E.M. values. **C** Volcano plot showing proteins enriched on ESD-EVPs (left) and ESA-EVPs (right). **D** C4A concentration determined by ELISA in HS (*n* = 8), ESA (*n* = 15) and ESD-derived EVPs (*n* = 15). **E** ELISA analysis of C4A on intact or lyzed EVPs (*n* = 6). *p < 0.05; **p < 0.01; ***p < 0.001, **** *p* < 0.0001. The data are expressed as mean ± S.E.M. values

We initially characterized following MISEV guidelines [11] EVPs isolated from pre-surgical plasma samples from patients with early-stage lung cancer with either a favorable prognosis (no recurrence after 5 years from surgery: Early-Stage Alive, ESA, *n* = 10) or unfavorable prognosis (deceased within two years from resection: Early-Stage Deceased, ESD *n* = 10) and disease free heavy smokers (HS). No significant differences in terms of size and number (Supplementary Fig. S1A), morphology (Supplementary Fig. S1B) and conventional EVP-markers (Supplementary Fig. S1C) were detected between the groups. WB analysis revealed the presence of tetraspanins and the co-isolation of lipoproteins (APOA1) (Supplementary Fig. S1D). Plasma-EVPs were positive for specific integrins described as having lung and liver-tropism (Supplementary Fig. S1E). Flow cytometry revealed that the majority of EVPs originated from platelets, endothelial and other stromal cells as demonstrated by high detection of lineage specific markers CD41b, CD42a, CD62P, CD31 and CD29 (Fig. 1A). ESD-EVPs presented higher levels of CD41b, CD42a, and CD31 compared to ESA-EVPs and HS-EVPs, suggesting potential differences in EVPs production related to patient prognosis. No significant differences were detected considering other markers (Supplementary Fig. S1F).

In a discovery set of 20 ESA and 20 ESD patients, we investigated whether ESD-EVPs differ from ESA-EVPs in terms of cargo composition focusing on miRNAs and proteins. Among 90 miRNAs detected, a differential representation of four miRNAs (hsa-miR-1307—3p, hsa-miR-451a, hsa-miR-29a-3p and hsa-miR-199a-3p) was found in the comparison between the two groups (Suppl. Table S1). Then, dPCR analysis conducted on a training cohort of 30 patients and 8 HS confirmed that ESD-EVPs were enriched in miR-29a-3p and miR-199a-3p content compared to both HS and ESA-EVPs (Fig. 1B). A non-statistical trend in the enrichment of miR-1307—3p and miR-451 was also detected (Supplementary Fig. S1G). Proteomic analysis revealed 9 overrepresented proteins in ESD versus ESA-EVPs (Fig. 1C and Suppl. Table S2). Among them, Surfactant Pulmonary-associated Protein B (SFTPB) and C4A were the only two proteins enriched in ESD and also in the comparison with HS-EVPs (Suppl. Table S3—4 and Supplementary Fig. S1H). ELISA confirmed that C4A was enriched in ESD compared to ESA and HS (Fig. 1D) and that it was mainly located on the surface of EVPs, as vesicle lysis did not change the amount of detected C4A (Fig. 1E). To demonstrate the mechanism of loading of C4A on the surface of EVPs we performed high-salt washing presuming that this step may disrupt electrostatic interactions. After this washing step, we observed a decrease of C4A on the surface of ESD-EVPs (Supplementary Fig. S2A). Furthermore, to further prove the passive adsorption of C4A on circulating EVPs, we isolated EVPs from Healthy Donors (HD) and incubated with the plasma from ESD patients depleted from their own EVPs. As illustrated in Supplementary Fig. S2B, after incubation we observed higher levels of C4A on the membrane of C4A from HD-EVPs. Furthermore, ELISA quantification of plasma of HD, HS, ESA and ESD patients depleted from EVPs revealed the presence of higher amount of free C4A in the circulation of patients (Supplementary Fig. S2C). All these data suggest that C4A is enriched in the plasma of ESD patients and passively binds to the vesicles.

### EVP -associated miR-29a and C4A identify early-stage lung cancer patients with unfavorable prognosis

To investigate the potential use of miR-29a and C4A as prognostic biomarkers we initially evaluated their performance in the training cohort of 30 LC patients and 8 HS controls. Using the minimum threshold value of miR-29a observed in ESD samples (342.51 copies/ul), we observed a sensitivity of 1.0 and a specificity of 0.60 in patient classification within the training set. Interestingly, the combination of mir-29a and C4A (minimum value in ESD, 12 ng/ml) improved the performance achieving a sensitivity of 1.0 and a specificity of 0.933.

To validate the performance of the biomarkers potentially identified in the study we used a prospective consecutive collection of plasma samples from early stage (stage I-II) LC patients undergoing surgery with curative intent at our Institution. Plasma samples of 89 patients with a median follow up of 41 months were therefore assessed for miR-29a and C4A expression.

Both biomarkers individually showed moderate ability to discriminate between deceased and surviving patients, which improved when the two markers were integrated into a combined index in both the training and validation set (Supplementary Fig. S3). ROC analysis in the validation set yielded an AUC of 0.72 (95% CI, 0.58–0.85) for miR-29 and 0.73 (95% CI, 0.58—0.88) for C4A, while the combined score achieved an AUC of 0.78 (95% CI, 0.65—0.91). At the optimal J cut-off, the combined model showed a sensitivity of 93.3% and a specificity of 56.8%, with corresponding PPV and NPV of 30.4% and 97.7%, respectively, indicating enhanced sensitivity and NPV compared to the single markers (Table 1).

**Table 1:** Diagnostic performance of individual and combined markers in the validation set

*Marker*	*Sensitivity (95% CI)*	*Specificity (95% CI)*	*PPV (95% CI)*	*NPV (95% CI)*
*miR-29*	86.7% (62.1–96.3)	51.4% (39.2–63.5)	26.5% (16.0–40.2)	95.0% (81.8–99.1)
*C4a*	86.7% (62.1–96.3)	62.2% (49.6–73.4)	31.7% (19.6–46.8)	95.8% (83.8–99.3)
*Combination*	93.3% (70.2–98.8)	56.8% (44.4–68.4)	30.4% (19.0–44.9)	97.7% (86.5–99.7)

### ESD-EVPs selectively trigger endothelial cell proinflammatory activation favoring neutrophil recruitment

**Fig. 2:**
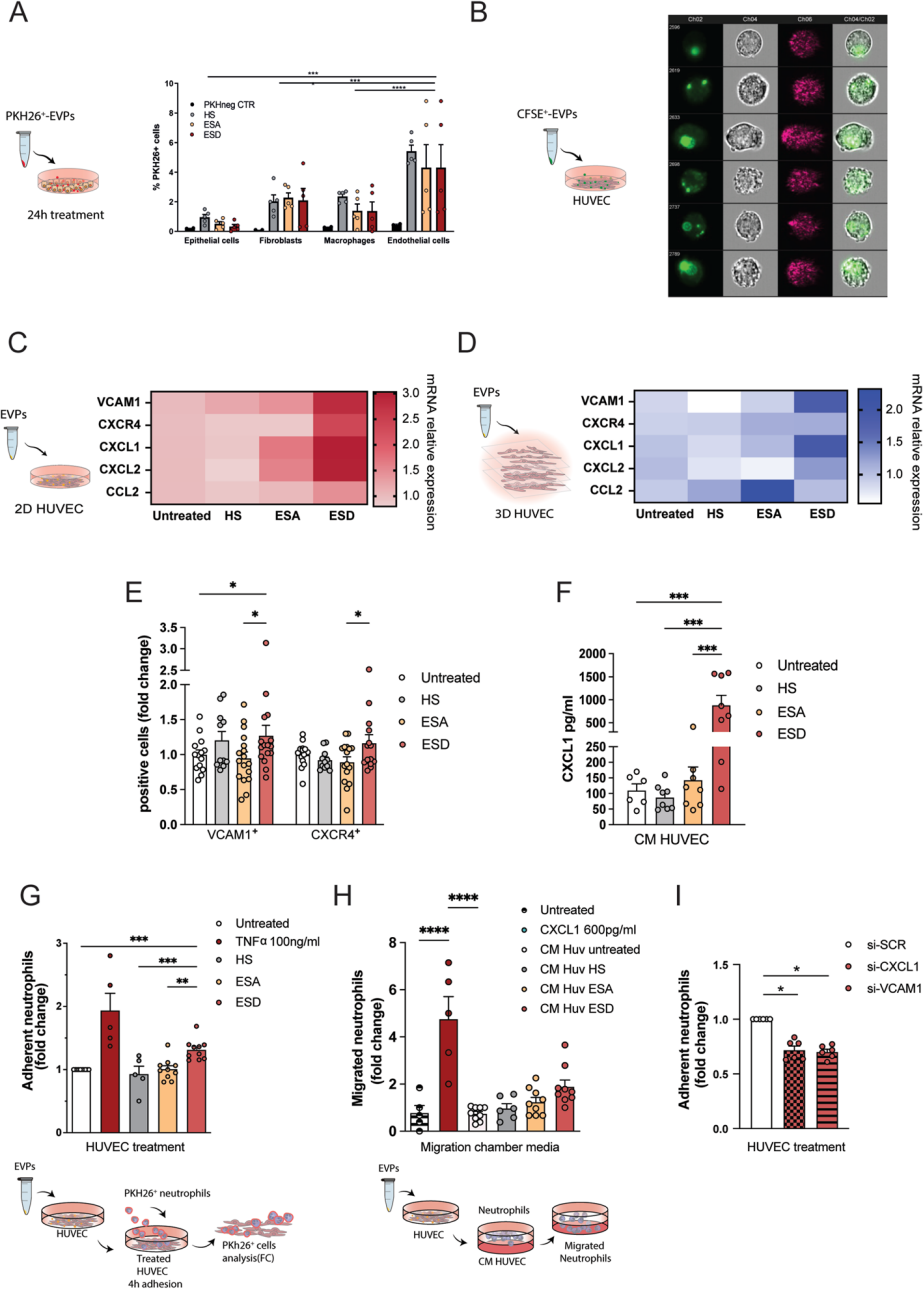
ESD- EVPs selectively trigger endothelial cell proinflammatory activation. **A** Flow cytometry analysis of the percentage of PKH26 + cells after treatment with PKH26-labeled EVPs (1 µg) (*n* = 5 per group). **B** Representative image of endothelial cells treated with CFSE labelled EVP and analyzed using Imagestream. Only green spots in focus inside the cells were considered positive events for the quantification. **C-D** Heatmap showing ESD-EVPs induced upregulation of VCAM1, CXCR4 and CXCL1 after 48 h of treatment as showed by qPCR analysis in 2D (**C**, *n* = 10 for ESA-ESD and *n* = 5 for HS) left) or 3D growth (**D**, *n* = 4, right). **E–F** Flow Cytometry analyses of the phenotype of EVP treated endothelial cells (**E**, *n* = 10) and CXCL1 (**F**) release by ELISA (*n* = 8). **G** Fold change of the number of adherent neutrophils on EVPs treated HUVEC (*n* = 9 for ESA-ESD and *n* = 5 for HS) compared to untreated cells (*n* = 9). TNF-α was used as positive control (*n* = 5). **H** Fold increase of migrated neutrophils towards HUVEC-treated CM (*n* = 8 for ESA-ESD and *n* = 6 for HS). CXCL1 (660 pg/ml) was used as control (*n* = 5). **I** Analysis of the fold change of neutrophils’ adhesion after ESD-EVPs treatment in silenced HUVEC cells (*n* = 6). Untreated cells were used as a control. *p < 0.05; **p < 0.01; ***p < 0.001, **** *p* < 0.0001. The data are expressed as mean ± S.E.M. values

Having demonstrated that ESD-EVPs differ in cargo content, we investigated their potential functional roles. To determine which microenvironment cells could be affected by these particles, we performed uptake experiments in vitro. As shown in Fig. 2A we noticed that EVPs are mainly incorporated by stromal cells rather than epithelial cells. By exploiting PHK26 dye labeled-EVPs we showed that endothelial cells possess the highest EVPs uptake, followed by macrophages and fibroblasts Efficient endothelial EVPs incorporation was validated by imaging flow cytometry analysis with carboxyfluorescein succinimidyl ester (CFSE) (Fig. 2B and Supplementary Fig. S4A).

Given the well-known role of the vascular compartment in PMN formation [4], the functional role of plasma-EVPs was investigated using HUVEC as recipient cells, both in conventional culture conditions and 3D bioprinted models (Supplementary Fig. S4B). ESD treatment induced a proinflammatory response in endothelial cells as demonstrated by transcriptomic analyses showing regulation of genes related to chemotaxis, cell migration and angiogenesis compared to ESA-EVPs (Suppl. Table S5). Higher expression of genes relative to endothelial activation and wound repair (EGR3, IL-16, CXCR4, CD34, CD40, TSPAN7 and KLF4) was observed together with down-modulation of genes related to vessel formation and suppression of oxidative stress (RND3 and ALKBH8; Suppl. Table S6). qPCR analysis of endothelial cells revealed the upregulation of inflammation-related genes such as *VCAM1, CXCR4*, *CXCL1, CXCL2* and *CCL2* upon ESD treatment (Fig. 2C-D). The upregulation of VCAM1 and CXCR4 was confirmed by Flow cytometry (Fig. 2E). Among the upregulated cytokines, only CXCL1 release was increased by ESD compared to HS or ESA treatments (Fig. 2F and Supplementary Fig. S4C). As VCAM1 and CXCL1 are closely related to immune and cancer cell recruitment, we wondered whether ESD-EVP could serve as gateways to immune cells during PMN formation. Thus, the interaction between EVP-stimulated endothelial cells and circulating myeloid cells (monocytes and neutrophils) was investigated using in vitro adhesion and migration experiments. As shown in Fig. 2G, ESD-EVP treatment of endothelial cells enhanced the adhesion of neutrophils but not of monocytes compared to controls (Supplementary Fig. S4D). A clear trend was observed in the migration of neutrophils towards CM from ESD-treated endothelial cells compared to CM from controls’ cells, although the difference was not statistically significant (Fig. 2H). Silencing of CXCL1 and VCAM1 in endothelial cells before ESD administration reduced neutrophils adhesion (Fig. 2I) demonstrating their central role in adhesion. Vessels sprouting and permeability in HUVEC after EVP treatment showed no difference between the two groups, suggesting that EVPs alone were not sufficient to induce vascular angiogenesis and leakiness (Supplementary Fig. S4E). This finding was corroborated by RNAseq results proving the downmodulation of well-known inducers of angiogenesis E2F7 and ETV5. To clear demonstrate that the endothelial activation is mediated mainly by EVs and no other contaminants present in our pellet, we performed sucrose density isolation on plasma of ESD patients separating sEV fractions from lipoproteins. As shown in Supplementary Fig. S4F, only the treatment with sEVs fractions recapitulated the observed upregulation of VCAM1 and CXCR4 in endothelial cells.

In conclusion, our results indicate that EVPs from ESD patients can modulate endothelial cells phenotype by increasing pro-inflammatory properties and immune cell recruitment.

### Endothelial cells stimulated by ESD-EVPs promote fibroblast activation through CXCL1

**Fig. 3:**
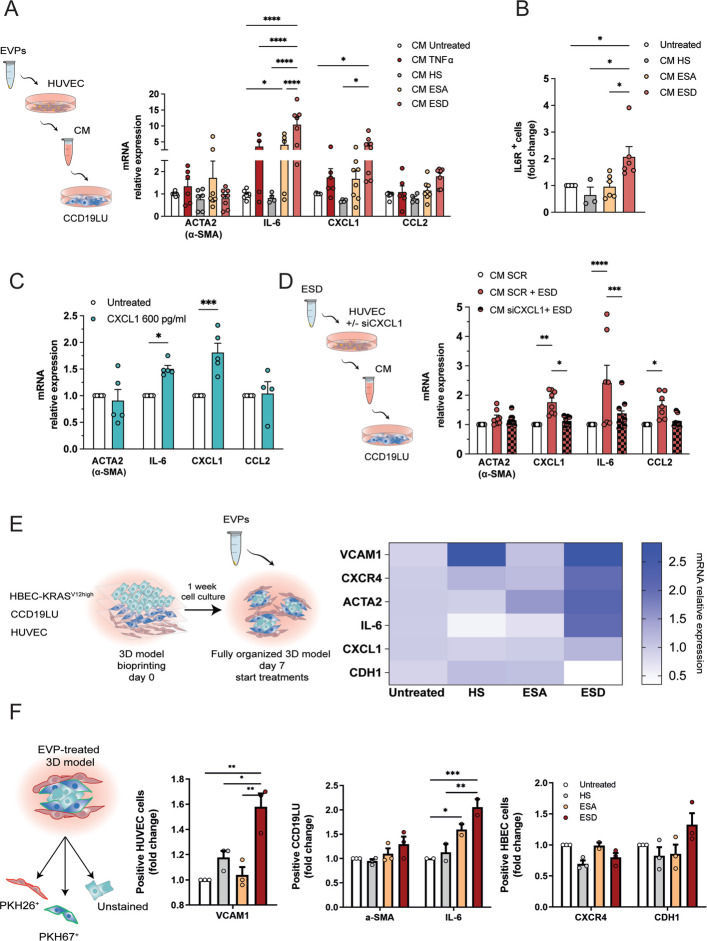
Endothelial cells stimulated by ESD-EVPs promote lung PMN formation. **A** Graph showing relative expression of fibroblast (CCD19LU) genes upon treatments with endothelial CMs. CM from untreated endothelial cells was used as control. Data are expressed as mRNA fold change relative to untreated cells (*n* = 8). **B** Fold change of IL6 receptor on the surface of CCD19LU treated with CM of endothelial cells compared to untreated cells by Flow Cytometry (*n* = 6) **C** The graph shows the relative expression of α-SMA, IL6, CXCL1 and CXCL2 in fibroblasts after 48 h of treatment with CXCL1 (600 pg/ml) (*n* = 6) **D** Histograms illustrated mRNA modulation of fibroblast treated with CM of endothelial cells silenced for CXCL1 and treated with ESD-EVPs (*n* = 8) **E**) Analysis of mRNA relative expression of several genes on 3D models treated with HS- ESA- and ESD-EVPs by qPCR. **F** Analysis of the percentage of VCAM1 +, α-SMA +, IL6 +, CXCR4 + and E-CAD + cells in multicellular 3D constructs after EVP administration. (*n* = 4 for each group). Untreated cells were used as a control. *p < 0.05; **p < 0.01; ***p < 0.001, **** *p* < 0.0001. The data are expressed as mean ± S.E.M. values

Phenotypic changes in fibroblasts, such as transition to a myofibroblast or inflammatory phenotype, are crucial factors during PMN formation [12]. Our investigation revealed that EVPs derived from LC patients did not directly influence the phenotype of normal lung fibroblasts (CCD19lu) as indicated by absence of significant differences in the expression of pro-inflammatory α-SMA, IL-6 and CXCL1 markers (Supplementary Fig. S5A). As fibroblasts can act as inflammation amplifiers when exposed to cytokine stimuli [13], we hypothesized that their activation might result from their crosstalk with other stromal cells. Fibroblasts were treated with CM obtained from endothelial cells treated with HS, ESA and ESD-EVPs. CM from untreated and TNF-α treated cells were used as controls. CM from ESD-treated endothelial cells led to significant upregulation of *IL6*, *CXCL1* mRNA and the expression of IL6 receptor on fibroblast’s surface compared to controls (Fig. 3A-B). Upregulation of IL6 was confirmed also in 3D models (Supplementary Fig. S5B). Since CXCL1 was the only cytokine that was consistently upregulated at both the gene and protein levels in ESD-stimulated HUVEC, we wondered if this protein could be responsible for the fibroblast modulation observed in ESD CM treatment. CXCL1 used at the same concentration as detected in ESD CM (600 pg/ml) strikingly upregulated *IL6* and *CXCL1* in treated fibroblasts (Fig. 3C). The central role of CXCL1 as a mediator of endothelial-fibroblast crosstalk was corroborated by the fact that CXCL1 silencing in ESD-treated endothelial cells abrogated CM ESD-induced fibroblast activation (Fig. 3D and Supplementary Fig. S5C).

In conclusion, CXCL1 produced by ESD-stimulated endothelial cells induces a positive feedback loop in fibroblasts by paracrine amplification of CXCL1 signaling resulting in the amplification of pro-inflammatory stimuli.

### ESD-EVPs treatment induces endothelial and fibroblast activation in a 3D heterotypic pre-metastatic niche model

Even though the lungs represent one of the main sites of lung cancer metastasis [14], lung PMN has been investigated starting mainly from other primary tumors; therefore, limited information is available about the role of EVPs in promoting metastasis in the lung. Since it has been described that activation of lung epithelial cells could be an important component of metastatic niche [15], to explore the effect of EVPs in a model recapitulating a lung PMN, we developed a 3D bioprinted-model, comprising endothelial cells (HUVEC), adult lung fibroblasts (CCD19lu) and human pre-neoplastic bronchial epithelial cells (HBEC-KRAS^V12high^). Owing to cell geometric confinement and spatial orientation within the fibers, this model promotes better differentiation and more functional cell organization, closely mimicking physiological cell growth conditions. Spheroidal structures with a defined cellular organization were observed after two weeks of culture (Supplementary Fig. S5D). These models were treated with ESA- ESD- and HS-EVPs or left untreated. qPCR in the multicellular 3D model showed that ESD-EVPs specifically upregulated *VCAM1* in 3D models (Fig. 3E). ESD treatment resulted in upregulation *of IL6* and *ACTA2* genes as a consequence of fibroblast activation. Flow cytometry analyses on cell subpopulation confirmed VCAM1 and IL6 upregulation in endothelial cells and fibroblast respectively, and revealed higher levels of E-cadherin on HBEC-KRAS after ESD administration compared to controls (Fig. 3F). DPCR on sorted cells unveiled that ESD treatments upregulated *VCAM1*, *CXCR4* and *CXCL11* in endothelial cells, *IL6* and *CXCL1* in fibroblast and *CDH1* in epithelial cells (Supplementary Fig. S5E). In conclusion, the 3D model corroborates previous data on ESD EVPs-induced endothelial activation and subsequent fibroblast phenotypic modulation.

### ESD-EVPs treatment promotes a proinflammatory and immunosuppressive pulmonary microenvironment

**Fig. 4:**
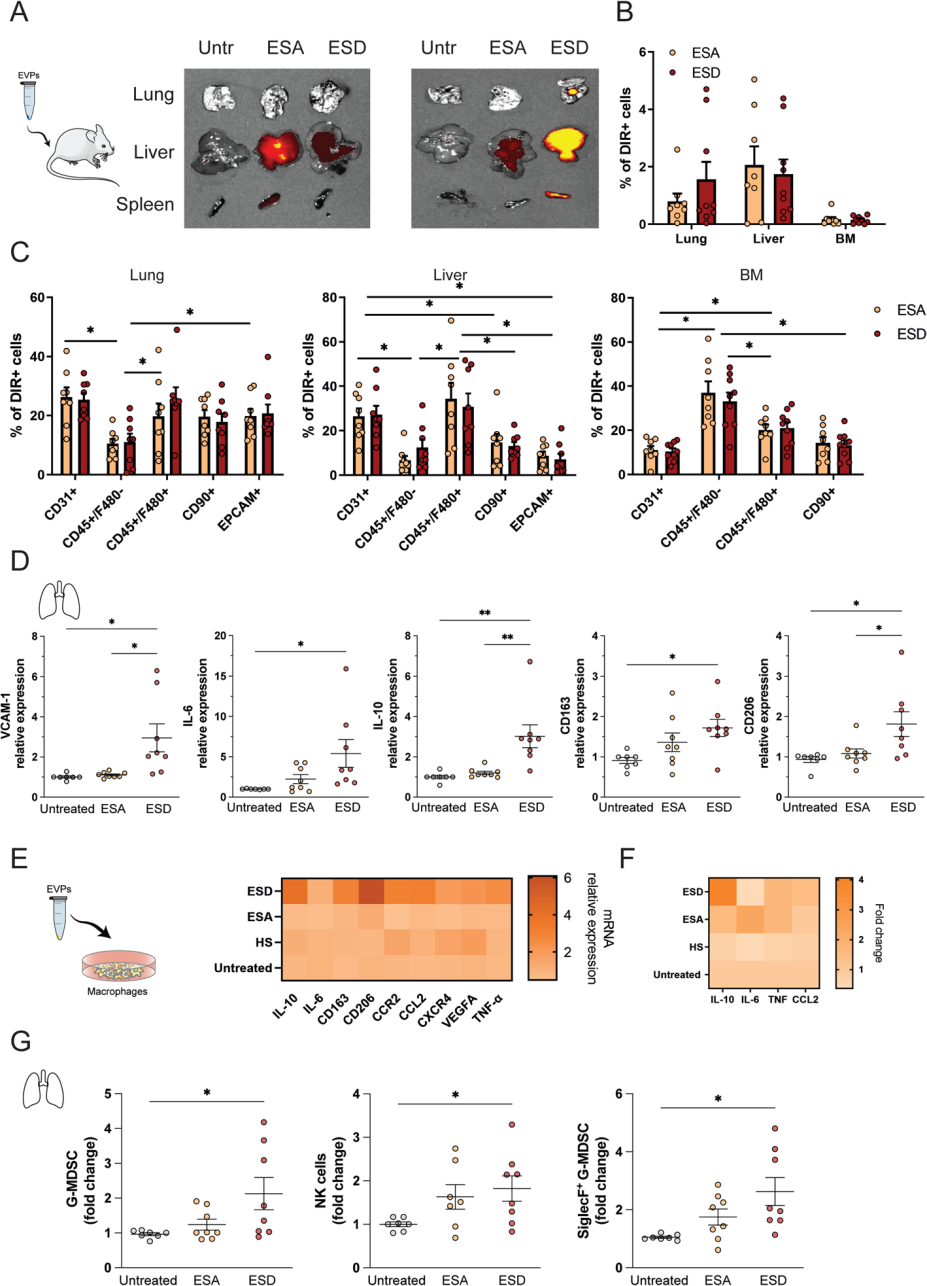
ESD treatment promoted a proinflammatory and immunosuppressive pulmonary microenvironment. **A** Representative images of ex vivo organs (lung, liver and spleen) showing the presence of EVPs (yellow–red signals). **B** Percentage of DiR + EVP in the lungs, bone marrow (BM) and liver of treated mice analyzed by Flow Cytometry. **C** Percentages of endothelial cells (CD31 +), leucocytes not macrophages (CD45 +/F480-), macrophages (CD45 +/F480 +), fibroblast (CD90 +) and epithelial cells (EPCAM +) among the DIR + cells detected in the lungs, liver and bone marrow of treated mice. **D** mRNA relative expression of vascular adhesion molecule (VCAM1), cytokines and receptors (IL6 and CXCR4) and macrophagic genes (IL10, CD206 and CD163) in the lungs of treated mice with ESA and ESD; untreated mice were used as control (*n* = 8 for ESA and ESD, *n* = 7 for untreated mice). **E–F** Heatmap showed the mRNA relative expression (**E**) or secreted proteins (**F**) of key genes of macrophages phenotype upon HS, ESA or ESD treatment. Untreated cells used as controls (*n* = 5 for each group). **G)** The graphs show the percentage of the different subsets of immune cells (only myeloid compartment) detected by FC in the lungs of mice upon EVP treatment and expressed as fold increase. Data are expressed as mean + S.E.M *p < 0.05; **p < 0.01; ***p < 0.001

Based on the assumption that several aspects of endothelial cell function are similar in humans and mice, including murine endothelial cells expression of the C4a receptor (PAR), and conserved miR-29a sequence, we set up an experimental system suitable to study the tropism of EVP, in vivo. Fluorescently labeled EVPs from early-stage lung cancer patients were delivered to mouse lungs through tail vein injection. As shown in Fig. 4A, after 24 h from *i.v*. injection, DiR^+^-EVPs were detectable in the lungs, liver and spleen. To confirm these results, lungs, liver and bone marrow (BM) were dissociated and analyzed by flow cytometry. The results confirmed the presence of EVP-DiR^+^ cells in both lungs and liver and at weak intensity in the BM (Fig. 4B). A greater EVP uptake by endothelial cells and macrophages both in lungs and livers was detected without difference between ESA and ESD-EVPs (Fig. 4C).

qPCR confirmed endothelial activation by ESD treatment, as evidenced by the upregulation of *VCAM1* and *CXCR4* in mouse lungs. In addition, upregulation of *IL6*, likely due to fibroblast activation, was detected. Increased transcription of the macrophagic activation marker *CD206*, macrophagic receptor *CD163* and *IL10* cytokines was noted, suggesting the induction of an immunosuppressive phenotype in macrophages by ESD treatment (Fig. 4D). A clear trend, although not significant, in the increase in *CXCL1* upon ESD-EVP administration was observed (Supplementary Fig. S6A). Interestingly, signs of pre-metastatic niche formation such as CXCL1 and CD206 upregulation was observed in the liver of ESD treated mice (Supplementary Fig. S6B).

In vitro experiments proved that ESD treatment of macrophages directly mediates the upregulation of both immunosuppressive and inflammatory genes as shown by qPCR analysis indicating upregulation of immunosuppressive markers such as *CD206*, *CD163, VEGFA, CCL2 and IL10* while *IL6* remained unchanged (Fig. 4E). Upregulation of proinflammatory markers such as *TNFA* and *CXCR4* was detected upon ESD treatments. The secretion of IL-10, CCL2 and TNF was confirmed by ELISA assays of CM of macrophages (Fig. 4F). In conclusion, our results indicated that ESD-EVPs directly modulate macrophage phenotype inducing a mixed phenotype expressing both immunosuppressive markers and inflammatory features.

Analysis of the immune compartment revealed that ESD-EVPs administration led to homing of granulocytic myeloid-derived suppressor cells (G-MDSCs expressing CD11b + Ly6G + Ly6Clow) and NK cells, whereas no significant difference was observed in the percentage of monocytes, in concordance to in vitro adhesion experiments (Fig. 4G and Supplementary Fig. S6C). Within the G-MDSC population recruited in the lungs, a higher abundance of sialic acid-binding Ig-like lectin SiglecF^+^ cells were observed in the ESD-treated mice compared to ESA-treated or untreated controls. In line with a possible activation of granulocytes by EVPs, the treatment of human normal density neutrophils with EVPs promoted the extrusion of extracellular traps, which was higher in the case of ESD EVs compared to ESA EVs (Supplementary Fig. S6D).

Collectively, these findings indicate that ESD-EVPs prime the PMN microenvironment, promoting an inflammatory and immunosuppressive milieu that in turn recruits G-MDSCs.

### ESD-EVPs pre-treatment promotes the homing and colonization of cancer cells in the lungs

**Fig. 5:**
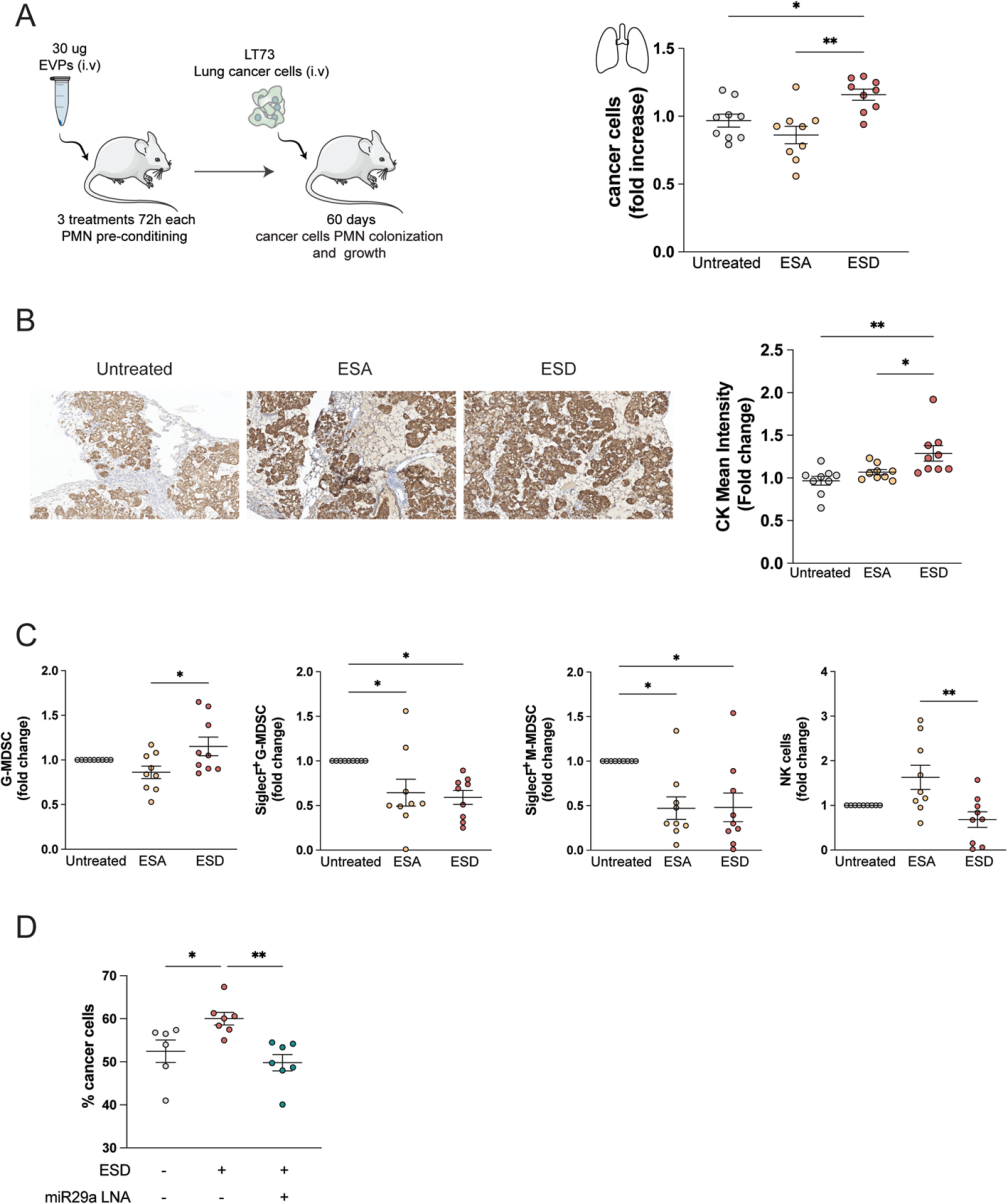
Lung cancer cells colonization in pre-treated lungs. **A** Graph showing the FC analysis of the amount of LT73 detected in the lungs of mice pretreated with EVPs at 45 days from *i.v.* injection. The values are expressed as fold increase (to untreated) of double negative cells (negative for 7AAD and murine MHC II) representing human cancer cells. **B** Representative images and analysis the amount of cytokeratin staining detected in IHC slides of the lungs. The graph shows the fold increase of cytokeratin staining quantified using ImageJ software. **C** Analysis of immune cells detected in the lung of mice after 45 days from the *i.v.* of cancer cells. The graphs show the percentage of the different subsets of immune cells (only myeloid compartment) detected by FC (ex vivo) and expressed as fold increase. **D** Graphs showing the number of cancer cells grown in the lungs after treatment with ESD or ESD transfected with LNA-29 (*n* = 7 for ESD and ESD + LNA-29; *n* = 6 for untreated). Untreated mice were used as control. Data are expressed as mean + S.E.M *p < 0.05; **p < 0.01; ***p < 0.001

Preconditioning experiments revealed that ESD-EVPs can modulate the lung environment, inducing both inflammation and immunosuppression creating a favorable milieu for the growth of metastatic cells. To investigate whether treatment with EVPs would result in facilitated engraftment and growth of cancer cells in vivo, 5 × 10^5^ LT73 lung cancer cells were injected *i.v.* in SCID mice after lung preconditioning with EVPs. After 45 days, a significantly higher abundance of cancer cells in the lungs of mice treated with ESD-EVPs was detected by flow cytometry and by IHC staining for pan-CK compared with mice treated with ESA-EVPs or non-conditioned mice (Fig. 5A-B and Supplementary Fig. S7A). The analysis of the immune compartment showed an increased number of G-MDSC that persisted after the engraftment and growth of cancer cells and down-modulation of SiglecF + and NK cells (Fig. 5C and Supplementary Fig. S7B). To investigate the functional role of previously identified miRNAs in ESD-EVPs in the induction of an effective PMN, we transfected miR-29a inhibitor in EVPs. Remarkably, miR-29a inhibition in ESD strikingly reduced their pro-metastatic effect as showed by the decrease of engraftment of metastatic cells in the lungs of treated mice underscoring the central role of this miRNA in PMN formation (Fig. 5D). Taken together, these findings suggest that ESD-derived EVPs can modulate the lung microenvironment, thereby enhancing and facilitating the homing and growth of lung cancer cells.

### EVP-delivered miR-29a combines with C4A to induce the expression of pro-inflammatory cytokines in endothelial cells

Building on the in vivo results, we mechanistically investigated whether miR-29a upregulated in ESD compared to ESA-EVPs, could be responsible for priming endothelial cells at the PMN by treating HUVEC endothelial cells with commercially available miRNA mimic oligonucleotides. Endothelial cells were transfected with miR-29a mimics (mim-29a) showing that the replenishment of this miRNA partially recapitulated the effects of ESD treatment (Supplementary Fig. S8A) by upregulating CXCR4, CXCL1, CXCL2, CCL2 and down-modulating SPARC expression in endothelial cells while the replacement of miR-199a did not affect their phenotype compared to negative sequence control (SCR). Thus, we investigated whether miR-29a contained in ESD-EVPs could be responsible for the pro-inflammatory phenotype observed in endothelial cells.

**Fig. 6:**
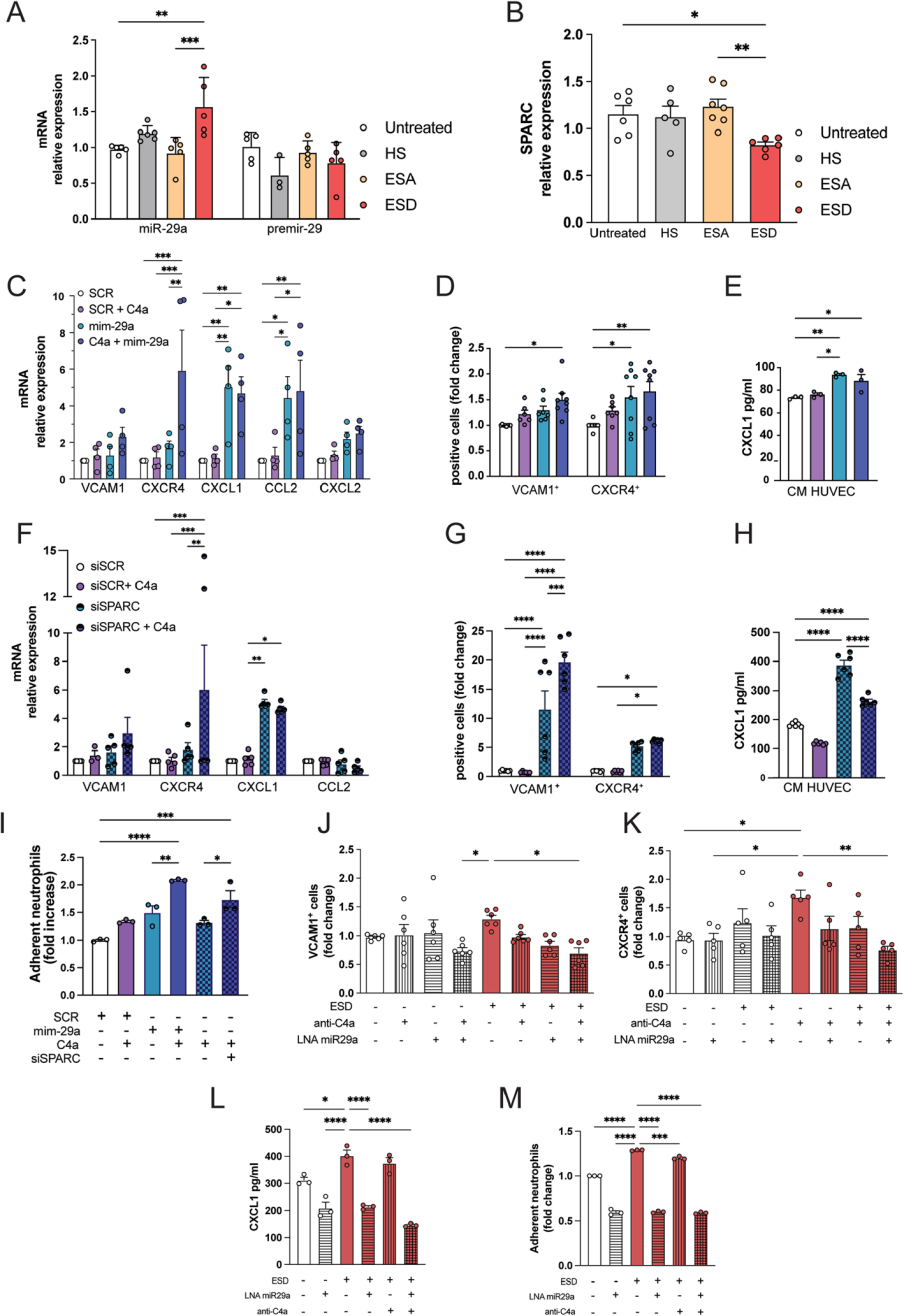
Combined effect of miR-29 and C4A through SPARC down-modulation. **A** mRNA relative expression of miR-29a and premir-29a upon EVP treatment. **B** Sparc downmodulation in HUVEC after EVP administration **C**) mRNA relative expression of VCAM1, CXCR4, CXCL1, CXCL2 and CCL2 on HUVEC cells treated with C4a, mim-29a and combination by qPCR. **D** FC analysis of VCAM1 + and CXCR4 + endothelial cells upon C4 and miR-29 treatment, expressed as fold increase to Scr treated cells **E**) Quantification of CXCL1 release of HUVEC treated with C4 and miR-29 alone or in combination by ELISA. **F-G-H** Modulation of endothelial cells phenotype after silencing of SPARC and C4 administration evaluated by qPCR (**F**), Flow cytometry (**G**) and ELISA (**H**). **I** The graph shows the number of PKH-26 + neutrophils attached to pre-treated endothelial layer treated with miR-29 mimic or siRNA-SPARC in combination with C4a analyzed by FC. **J-K** Flow cytometry analysis of VCAM1 + (**J**) and CXCR4 + (**K**) endothelial cells after silencing of miR-29 (LNA-29) and blocking of C4 by antibody (anti-C4a) and concomitant treatment with ESD-EVPs, expressed as fold increase to Scr treated cells. **L-M** Secreted CXCL1 quantification (**L**) and measurement of adherent neutrophils (**M**) on endothelial cells after silencing of miR-29 (LNA-29) and blocking of C4 by antibody (anti-C4a) and concomitant treatment with ESD-EVPs. Transfected cells with negative sequence control (scr) were used as a control. Data are expressed as mean + S.E.M *p < 0.05; **p < 0.01; ***p < 0.001; ****p < 0.0001

As shown in Fig. 6A, we observed that miR-29a was effectively transferred by ESD-EVPs to endothelial cells as demonstrated by its upregulation in recipient cells after ESD administration, while the expression of the precursor (premiR-29a) remained unchanged. The effectiveness of this miRNA on recipient cells was demonstrated by down-modulation of *SPARC*, a known target of miR-29a (Fig. 6B). We concluded that miR-29a transferred from EVPs to endothelial cells could be functionally involved in their pro-metastatic priming. Since the transfer of miR-29a alone did not fully recapitulate the proinflammatory phenotype of endothelial cells upon ESD treatments, concerning VCAM1 upregulation, we further investigated its potential synergism with the complement protein C4A also found upregulated in ESD samples (Fig. 1C). First, we investigated the presence of the receptors for C4A (PAR1 and PAR4) on endothelial cells observing that only PAR4 is expressed on the surface of recipient cells (Supplementary Fig. S8B). Transcriptomic analysis revealed that concomitant miR-29a replacement and C4A administration had a combined effect on endothelial cells demonstrated by the up-regulation of 604 genes compared to 516 with miR-29a alone. Pathways’ enrichment analysis highlighted that these upregulated genes were related to response to chemokine, neutrophil migration and cellular chemotaxis (Suppl.Table S7).

We confirmed that the combination treatment effectively recapitulated the proinflammatory phenotype induced by ESD treatment, as demonstrated by increased of mRNA expression (Fig. 6C) and elevated protein levels of VCAM1, CXCR4 (Fig. 6D) and CXCL1 (Fig. 6E). Among the potential target of miR-29a, RNAseq and qPCR showed that SPARC was downregulated after miR-29 mimic transfection (Supplementary Fig. 8 C and Suppl. Table S8).

To assess the key role of SPARC in the observed effects, we silenced SPARC in endothelial cells alone or in combination with C4 administration (Supplementary Fig. S8D). We observed VCAM1, CXCR4 and CXCL1 upregulation both at mRNA (Fig. 6F) and protein level (Fig. 6G), after SPARC silencing and C4 treatment. C4A in combination with miR-29a mimic or SPARC silencing increased the attachment of neutrophils on endothelial cell layers (Fig. 6I).

Blocking C4A (neutralizing Ab, anti-C4A) was sufficient to block VCAM1 increase and this effect was potentiated by combinatory treatment with miR-29a inhibition (LNA-29a) (Fig. 6J). Concomitant blockade of C4A on EVPs and miR-29a transfer inhibits CXCR4 up-regulation induced by ESD-EVPs (Fig. 6K). MiR-29a alone appeared to be responsible for CXCL1 upregulation, as treatment with C4A did not affect CXCL1 levels (Fig. 6L) or neutrophil adhesion (Fig. 6M).

Such conclusive evidence showed that the interplay between miR-29a and C4A plays a pivotal role in mediating microenvironmental alterations induced by ESD- EVPs leading to the generation of proficient niche for metastasis formation.

## Discussion

Lung cancer is a highly metastatic cancer and the majority of LC deaths are attributable to the high incidence of regional and distant metastasis [16]. Studies show that synchronous lung metastases most often originate from lung cancer (41%), followed by colorectal (10%), kidney (7%), pancreatic (7%), and breast (6%) cancers [17]. At diagnosis, lung cancer metastases primarily occur in the opposite lung (49–59%), then the brain (30–42%), bone (29–39%), liver (13–26%), and adrenal glands (10–24%) [18]. Rapid intervention with complete resection of the primary tumor in early stage NSCLC patients ameliorates the prognosis, even if 30—55% of these patients experience recurrence and metastasis within a few years from the diagnosis [19].

EVPs released during cancer progression play a central role in metastasis by reaching and modifying distant microenvironment to create a favorable “soil” for cancer cell homing and outgrowth, called PMN [20].

While the role of tumor-secreted EVPs has been extensively described, we show here for the first time the involvement of systemically circulating vesicles in the formation of lung PMN. We observe that ESD-EVPs exhibited a different cargo composition and higher levels markers associated with platelets and endothelial cells (CD41b, CD42a, and CD31) compared with HS and ESA. These markers are involved in platelet activation mediating their binding to leukocytes and endothelial cells [21]. Elevated levels of platelet-derived EVPs have been linked to disease progression and reduced overall survival in NSCLC patients [22].

Here, we demonstrate that early changes in circulating EVP are detectable at the initial stages of the disease and may reflect very early cancer-related systemic alterations. The presence of such early systemic EVP modifications raise important questions about their mechanistic contributions to PMN formation. Our data demonstrate that endothelial cells are the primary cellular recipients of circulating EVPs, followed by macrophages, in both in vitro and in vivo settings. This preferential uptake pattern is consistent with previous reports showing that circulating EVPs can roll, arrest and accumulate on vascular endothelium and are predominantly internalized by endothelial cells and macrophages across species [23].

Recent findings revealed that lymphatic endothelial cells in lymph nodes PMN serve as primary recipients of melanoma-derived EVPs, while macrophage incorporation occurs with delayed kinetics and reduced efficiency. This preferential endothelial uptake resulted in phenotypic alterations that promoted PMN formation [12]. In line with these findings, our functional analyses revealed that circulating EVPs in patients with poor prognosis trigger endothelial cell inflammatory responses, upregulating the adhesion molecule VCAM1 and enhancing cytokine production in different in vitro and in vivo models. This endothelial activation represents a previously uncharacterized mechanism driving microenvironment conditioning during PMN formation. We showed that CXCL1 released by ESD-EVPs-treated endothelial cells activate fibroblasts, by promoting *CXCL1* and *IL6* transcription and establishing a paracrine signaling cascade. Given the established roles of CXCL1 and IL6 in immune cell recruitment and modulation, this endothelial-fibroblast crosstalk likely facilitates immune cell infiltration into lung PMN. Consistent with this hypothesis, ESD-EVP treatment enhanced G-MDSC homing to the lungs, specifically enriching for a Siglec-F-positive neutrophil subpopulation with potent immunosuppressive and pro-angiogenic properties implicated in lung cancer progression [24]. Indeed, G-MDSCs are involved in immunosuppression and are linked to PMN formation [14, 25–28]. Adhesion assays revealed that neutrophil binding was enhanced in ESD-EVPs treated endothelial cells compared to ESA or HS, likely due to VCAM1 upregulation, which could account for the higher G-MDSC accumulation observed in the lungs of ESD-treated mice [29]. We aknowledge the limitation of our study regarding distant organ colonization. Indeed, with our experimental model (primary cell line LT73) we do not observe major liver, bone or brain colonization and we have focused our investigation on the promotion of PMN within the lungs themselves. Lung tropism and metastatic outgrowth also requires euthanizing mice to avoid suffering preventing therefore longer observation time for investigation of potential liver or bone PMN for which we have correlative data. Our in vivo experimental model clearly lacks of adaptive immunity, and this represents a limitation of the study. However, we know, based also on literature [5, 30], that in the early stages of pre-metastatic niche formation, myeloid cells such as neutrophils and NK cells play a crucial and fundamental role, thereby strengthening our findings. Along with its biological significance, the possibility of targeting this mechanism could be highly clinically relevant, as VCAM1 blockage has been shown to prevent neutrophil infiltration in melanoma lung metastasis [29]. Our data indicate that activated fibroblasts in response to endothelial signals can contribute with additional chemotactic signals, upregulating cytokine and chemokine expression thus creating a multi-cellular recruitment network within the evolving PMN.

Mechanistically, the proinflammatory endothelial response induced by ESD-EVPs results from synergistic interactions between miR-29a and C4A cargo within these vesicles. We demonstrated that ESD-EVP-transferred miR-29a promotes cytokine transcription in recipient cells, partially recapitulating the ESD-induced activation phenotype. While the role of miR-29a in endothelial biology remains incompletely characterized, emerging evidence supports its regulatory functions in endothelial homeostasis [31, 32]. However, miR-29a alone was insufficient to fully reproduce ESD-EVP effects, indicating additional contributory factors. Among the proteins enriched in ESD-EVPs, we identified C4A, a complement family member and isotypic variant of C4 [33] with established roles in innate immunity and microbial defense [34]. Notably, C4A has been implicated in supporting carcinogenesis [35] and metastatic progression [36, 37], suggesting context-dependent functions beyond immune surveillance. Our data reveal that C4A and miR-29a act synergistically to fully recapitulate the ESD-induced endothelial phenotype, identifying a novel molecular mechanism underlying EVP-mediated pre-metastatic niche conditioning.

MiR-29a blocking reduced metastasis without altering primary tumor growth, suggesting that miR-29a function is prevalently linked to remote tissue conditioning rather than local tumor promotion [38]. Interestingly, depletion of the putative target gene of this miRNA named secreted protein acidic and rich in cysteine (SPARC) has been shown to induce TME remodeling [39] or lung inflammation in mouse models (Sparc −/−) [40]. The same authors found signs of endothelial damage in tumors of Sparc −/− mice [41] suggesting a possible role of SPARC in the modulation endothelial inflammatory features. Interestingly, SPARC down-modulation in the TME was associated to increased neutrophil recruitment and extracellular traps extrusion. Moreover, since miR-29a has multiple targets we are aware that SPARC may be not the only mediator of endothelial activation in ESD patients.

Our findings translate miR-29 and C4A into clinically relevant biomarkers to identify early stage patients with unfavorable prognosis as tested in both training and validation cohorts. Since our data revealed the potential involvement of platelets and their EVs in PMN formation, these results also open the perspective to investigate stratification of early stage patients for the adoption of preventive therapies using anti-platelets drugs alone or in combination with standard therapy on the basis of miR-29a and C4A levels.

Beyond miR-29a, ESD-EVPs from patients showed higher levels of miR-199a, which has been independently associated with NSCLC presence [42] along with C4A. The enrichment of C4A likely reflects cancer-induced complement cascade activation, as lung cancer patients exhibit elevated complement split products in biological fluids [13]. Considering a potential translation of our findings into a therapeutic setting, several strategies were developed considering miRNAs replacement using nanoparticles in cancer. However, although microRNAs modulation showed promising results in pre-clinical models, the clinical use of these small RNAs is limited by several challenges such as toxicity, off-targets effects in non-tumoral tissues and induction of unwanted immune responses [43]. Furthermore, for cancer treatment, the use of complement inhibitors is still far from a direct application to cancer patients for the risk of the development of severe infections [44].

## Conclusions

Collectively, our findings demonstrate that circulating EVPs in early-stage lung cancer patients function as both predictive biomarkers and functional mediators of systemic disease progression. We demonstrate that EVP-induced endothelial activation triggers a multi-cellular cascade involving fibroblast stimulation and immunosuppressive cell recruitment, collectively generating a PMN fostering lung tumor cell colonization and metastatic outgrowth. The identification of miR-29a and C4A as synergistic mediators of this process can have potential therapeutic implications, since interfering with these pathways could disrupt PMN formation. These discoveries not only advance our understanding of the earliest steps during lung cancer metastatic progression but suggest that EVP-based liquid biopsies could allow a better clinical management of these patients with interventions during the pre-metastatic window, when therapeutic efficacy may be optimal.

## Methods

### Patients cohort and sample collection

Plasma EVPs were obtained from samples collected before surgery from patients with resectable early-stage (I-II) lung cancer. The discovery cohort was divided into patients with early-stage lung cancer who died within two years from diagnosis (ESD; *n* = 20), and patients alive at five years post-diagnosis (ESA; *n* = 20). Biomarkers were validated using plasma samples from our biorepository series of 30 patients (training cohort) with matching clinicopathological characteristics. Control plasma samples from 8 heavy smokers (HS) aged 50—75 years with minimum 30 pack-year index belonging to the Low Dose Computed Tomography (LDCT) screening trial BioMild (ClinicalTrials.gov: NCT02247453) [45] were also included. For the validation cohort we consecutively enrolled 89 early-stage lung cancers patients with their clinical information and a median follow-up (FU) of 41 months. Patient characteristics are summarized in Suppl. Table 9.

Peripheral blood (5—10 ml) was collected in K_2_EDTA tubes and processed within 2 h to minimize pre-analytical variables. Plasma was obtained by double centrifugation at 1,258 × g for 10 min at 4 °C to remove cellular debris and stored at −80 °C until analysis. The study was approved by the Institutional Review Board (INT 133/19, INT 0021/11) with written informed consent from all participants.

### EVP isolation and quantification

EVPs were isolated from 1 ml plasma diluted to 4.2 ml with sterile filtered PBS 1X to ensure proper tube filling. Samples were ultracentrifuged using a TLA-100.3 fixed-angle rotor at 120,000 × g for 90 min at 4 °C in TL-100 ultracentrifuge (Beckman Coulter, Brea, CA, USA). Supernatant was carefully removed, and the EVP-enriched pellet was washed with 1 ml of filtered PBS at 120,000 × g for 60 min at 4 °C to remove residual contaminants. The final EVP-enriched pellet was resuspended in 200 μl of sterile filtered PBS. After the protein quantification single-use aliquots of 15 μg protein EVPs were prepared and stored at −80 °C to avoid freeze–thaw cycles.

### EVP Characterization

#### Protein quantification

EVP protein content was determined using Bradford assay (Sigma-Aldrich) with BSA standards (0—2 mg/ml in PBS). EVP samples (2 μl) and standards were mixed with Bradford reagent (198 μl) in 96-well plates and absorbance measured at 595 nm using a Tecan Infinite M1000 plate reader. Protein concentrations were calculated by interpolation with the standard curve (R^2^ > 0.99). Protein yield was calculated as μg of proteins/ml of the original plasma.

#### Nanoparticle tracking analysis

EVP concentration and size distribution were determined using NanoSight NS300 (Malvern Panalytical). Briefly, 2 μl of 15 μg EVP aliquot was diluted in PBS to 1 ml final volume. Three 30-s videos were recorded per sample with camera level 15/16 and detection threshold 2—7. Particle size and concentration were analyzed with NTA 3.2 software.

#### Western blot analysis

EVPs (45 μg) were lysed in RIPA buffer supplemented with protease inhibitors (Sigma-Aldrich, 1:100) for 30 min at 4 °C. Proteins were separated using Bolt™ Bis–Tris Plus 4—12% gels (Thermo Fisher Scientific) and transferred to PVDF membranes using an iBlot™ Gel Transfer Device (Thermo Fisher Scientific). After blocking with 5% non-fat milk, membranes were incubated overnight at 4 °C with primary antibodies: anti-CD9, anti-CD81, anti-TSG101, anti-ApoA1 and anti-GM130 (all Cell Signaling), followed by appropriate secondary antibodies (GE Healthcare, 1:2000) for 1 h at RT. Signals were detected by enhanced chemiluminescence using Amersham ECL Prime kit and a MINI HD9 imaging system (Cleaver Scientific).

#### Transmission electron microscopy

The investigation of EVPs through negative staining was conducted as previously described [46]. Briefly, 5 μg of EVPs were placed onto glow-discharged 200-mesh formvar/carbon copper grids (Electron Microscopy Sciences). After two washes in H2O, EVPs were fixed in 2.5% glutaraldehyde. Following two additional washes in H_2_O, the samples were stained with 2% uranyl acetate for 1.5 min. Negative-stained samples were examined using a digitized Talos L120C electron microscope (Thermo Fisher Scientific) at 120 kV with a CCD camera.

### EVP labeling

For uptake assays, 15 μg EVPs were labeled with PKH26 (Sigma-Aldrich) in 1 ml PBS for 5 min at RT, washed by ultracentrifugation (120,000 × g, 60 min), and resuspended in 150 μl PBS. As a negative control, the same amount of PKH26 in 1 ml PBS without EVP was ultracentrifuged and resuspended as above.

For imaging flow cytometry, 30 μg of EVPs were labeled with CFSE (50 μM, ThermoFisher) for 2 h at 37 °C following the protocol described by Morales et al [47]. For biodistribution studies, EVPs were labeled with DiR (1—2 μM, Biotium) for 20 min at RT. All labeling included ultracentrifugation washing to remove unbound dye.

### Cell treatments

#### EVP treatments

For all in vitro treatments with plasma-derived EVPs, 5 × 10^4^ cells/well (CCD19lu, HUVECs, or macrophages) were seeded in 6-well plates and treated with 15 μg of EVPs for 48 h. Untreated cells were used as controls.

For conditioned medium (CM) collection, complete medium was replaced with 1 ml RPMI-free medium (without serum and antibiotics) 24 h after the treatments in order to avoid contamination due to growth factors present in the complete medium. CM was collected after 24 h, centrifuged at 1,200 × g for 10 min to remove debris and used immediately or stored at −20 °C.

For uptake experiments, cells were treated with 1 μg PKH26^+^ EVPs for 24 h. The number of PKH26^+^ cells was determined by Flow Cytometry. For imaging flow cytometry, 1 × 10^5^ cells were seeded and treated with 15 μg of CFSE labelled EVPs. After 24 h, the cells were harvested, pelleted, and analyzed with a Cytek® Amnis® ImageStream®X Mk II imaging flow cytometer.

#### Fibroblast treatments with HUVEC CM

CCD19lu cells were seeded as previously described. After 24 h of culture, the cells were treated with 1 ml of EVP-treated HUVEC CM, RPMI FREE + CXCL1 600 pg/ml (Peprotech) and RPMI FREE alone as control. All treatments were performed for a total of 48 h.

#### Transfection and recombinant protein treatments

HUVECs at 60—80% confluence were transfected with mirVana™ miRNAs (miR-199a-3p, miR-1307—3p, miR-29a-3p, scrambled control; 5 nM) using Lipofectamine 2000 (Thermo Fisher Scientific) according to manufacturer's protocol. After 4 h, medium was changed to complete medium for 48-h incubation. For C4A treatments, recombinant C4A (300 nM, Origene) was used either alone or in combination with miRNA mimics.

### Functional assays

#### 3D bioprinting experiments

Multicellular lung constructs were generated using extrusion-based 3D bioprinter equipped with a previously described [48, 49] microfluidic-based printing head (MPH), integrated with a coaxial needle for precise spatial deposition. Briefly, three distinct bioinks, each containing a single cell type—HUVEC-PKH-26^+^, CCD19lu-PKH-67^+^ and HBEC-KRAS^V12high^—were deposited in an alternating sequence every four layers, from bottom to top, to assemble a multicellular 12-layer construct (8 × 8 × 1 mm^3^). Each bioink was formulated with 0.8% (w/w) PEG-fibrinogen (PF) and 4% (w/w) low molecular weight alginate (LMW-ALG, Mw 33 kDa). Irgacure 2959 was used as radical photoinitiator at a concentration of 0.01% (w/w).

After UV crosslinking (365 nm, 4–5 mW/cm^2^) for 5 min, the bioprinted constructs were cultured for 7 days to allow the proper 3D cellular organization, and subsequently treated with EVPs (15 μg) administered twice at 48 h intervals. Then, the samples were either directly fixed for immunofluorescence analysis or enzymatically digested using collagenase type 2 (250 U/ml), collagenase D (0.5 U/ml) and Alginate Lyase (0.4 U/ml). The resulting cell suspension was used for dPCR, FC, and RNAseq analyses.

### In vivo experiments

For all the in vivo experiments, 7–12 weeks old SCID female mice (Charles River Laboratories) received intravenous (i.v.) injections of 30 µg of EVPs every 72 h for three times.

For the biodistribution and pre-conditioning experiments, mice were fed with AIN 93G diet for 4 days before the experiments. Dir + EVPs were administered intravenously 24 h before imaging. Then, mice were anesthetized in an induction cage with 5% isoflurane in 100% oxygen at 1.5–2 L/min until the mouse is fully anesthetized (confirmed by absence of response to toe pinch). Mice were transferred into the IVIS imaging chamber and connected to the nose cone delivering 1.5–2% isoflurane for anaesthesia maintenance. Images were acquired using Spectrum IVIS. After imaging, mice were euthanized and organs recovered for ex vivo IVIS imaging (lungs, liver and spleen) maintaining the same acquisition settings. the mice were sacrificed 24 h after the last treatment.

For the colonization assay, following lung pre-conditioning, 5 × 10^5^ LT73 cells were injected i.v. (*n* = 10 mice per group) and the mice were monitored for 60 days. At the conclusion of the in vivo experiments, lungs were harvested for IHC, flow cytometry and qPCR analysis. All procedures followed Ethics Committee guidelines (OPBA 454/2021-PR).

### Molecular biology

#### Nanostring analysis of EVP-miRNAs

For NanoString analysis, 90 µg of EVPs were processed for RNA extraction following the same methodology as previously described, but with the addition of a spike in sequence (osa-miR-414) during the lysis process. After RNA quality check, the analysis of a panel of approximately 800 different miRNAs (Human v3 miRNA Assay: 827 miRNAs, 5 mRNAs, and 25 internal reference controls, nCounter) were performed following manufacturer’s instruction.

##### Computational analysis of nanostring data

The raw data were processed using geometric mean cartridge correction, mean background correction, TGM normalization and non-parametric batch effect adjustment. To identify genes differentially expressed between the different statuses (dead or alive) after accounting for differences in expression among cartridges, an A + B ANOVA for fixed-effects models was applied. Statistical significance was set at *p* < 0.05.

#### EVPs proteomic analysis

Proteomic profiling of plasma EVPs was performed by Tymora Analytical (West Lafayette, United States) as previously described [50, 51]. Starting with 100 µl of plasma, optimized protein extraction and double digestion with Lys-C and trypsin were performed. The samples were then desalted and analyzed by Q-Exactive HF-X MS coupled with an Ultimate 3000 LC. This system allowed for the detection of 500—1,500 unique proteins in each sample.

EVPs protein profile bioinformatic analysis was then conducted as follows: proteins contained in ESD vs. HS and ESA EVPs were analyzed using a comprehensive pipeline. This process began with data normalization using quantile normalization, which ensures that the distributions of intensities are comparable across samples [52]. Following normalization, a log2 transformation to stabilize the variance and improve the data interpretability was applied. Then LIMMA R package was used to perform differential analysis, fitting a linear model to the data and calculating contrasts to identify significant changes [53]. In particular, log2 Fold Change > 1 or log2 Fold Change < −1 and p-value < 0.05 were considered as threshold for the selection of the most variable features between classes. R software (version 4.2.3) was used for the analysis and plots.

#### RNA sequencing

##### RNA extraction and sequencing libraries

RNA was extracted from dry cell pellets using ReliaPrep RNA Miniprep Systems (Promega), following manufacturer’s instructions. Quality check was performed with a 4150 TapeStation System RNA Screen Tape (Agilent), Qubit RNA Broad Range assay (Thermo Fisher Scientific) and a Nanodrop measurement. Sequencing libraries were constructed from the extracted total RNA using the QuantSeq 3' mRNA-Seq Library Prep Kit FWD (Lexogen) following the manufacturer’s instructions. Quality check was performed with 4150 TapeStation System D1000 HS assay (Agilent) and Qubit DNA HS assay (Thermo Fisher Scientific). Libraries were equimolarly pooled at 4 nm and sequenced on NextSeqSystem (Illumina).

##### Bioinformatic analysis

After sequencing, FASTQ files were imported into the Kangooroo Platform by Lexogen for quality controls and alignment. Raw gene counts were then obtained. For both the cell line and the 3D model, RNA-seq analysis of the cell line was performed using the edgeR Bioconductor/R package (version 3.42.4). Low-expression genes were filtered using the filterByExpr function and normalization was carried out with the TMM method. For the cell lines, differential expression analysis (DEA) was conducted using a Generalized Linear Model (GLM). Six comparisons were performed: (1) ESD vs ESA, (2) ESA vs NT, (3) ESD vs NT, (4) C4 + mim-29 vs scr, (5) C4 vs scr, and (6) mim-29 vs scr. For each comparison, we set a log fold change (logFC) > = 1 or < = −1 and False Discovery Rate (FDR) < 0.05 to identify significantly differentially expressed (DE) genes. To explore the biological functions of the DE genes, we conducted Gene Ontology (GO) enrichment analysis using the clusterProfiler package from Bioconductor/R (version 4.8.3). Only GO terms and pathways with an adjusted p-value < 0.05 were considered significant. We also identified which DE genes from comparisons (1) mim-29 vs scr and (2) C4 + mim-29 vs scr are targets of the miRNA hsa-miR-29a-3p (MIMAT0000086), using the multiMiR Bioconductor/R package (version 1.28.0). For the 3D model, normalized counts were transformed into log2 counts per million (logCPM) for downstream analysis. The following comparisons were performed: (1) HBEC_ESD vs HBEC_ESA, (2) HBEC_ESD vs HBEC_HS, (3) HBEC_ESD vs HBEC_NT, (4) HBEC_ESA vs HBEC_HS, (5) HBEC_ESA vs HBEC_NT, (6) HBEC_HS vs HBEC_NT, (7) HUVEC_ESD vs HUVEC_ESA, (8) HUVEC_ESD vs HUVEC_HS, (9) HUVEC_ESD vs HUVEC_NT, (10) HUVEC_ESA vs HUVEC_HS, (11) HUVEC_ESA vs HUVEC_NT, (12) HUVEC_HS vs HUVEC_NT, (13) FIBRO_ESD vs FIBRO_ESA, (14) FIBRO_ESD vs FIBRO_HS, (15) FIBRO_ESD vs FIBRO_NT, (16) FIBRO_ESA vs FIBRO_HS, (17) FIBRO_ESA vs FIBRO_NT and (18) FIBRO_HS vs FIBRO_NT. Gene expression differences were evaluated by calculating log2FC values. Genes with log2FC ≥ 1 or ≤ −1 were identified genes with substantial expression variation.

### Statistical analyses

GraphPad Prism 9 (GraphPad Software Inc., CA, USA) was used to analyze both the in vitro and in vivo experiments. Statistical significance was determined using ANOVA with Tukey’s multiple comparison tests and unpaired or paired t-tests. Statistical significance was set at p < 0.05. All experiments were performed at least in triplicate, and the data are expressed as mean and standard error of the mean (SEM).

Raw data of the training and validation sets were log2-transformed to improve distribution properties for statistical and computational analyses. Both biomarkers (miR-29a and C4a) were then standardized to z-scores, within each set, to harmonize their scales and reduce the impact of measurement variability. A combined index was generated as the weighted sum of the two standardized markers, where the weight (w) corresponded to its fold-change between deceased and surviving patients in the training set. The resulting continuous values for miR-29, C4a, and the combined score were used to generate receiver operating characteristic (ROC) curves and calculate the respective areas under the curve (AUCs) with 95% confidence intervals (CIs). Sensitivity, specificity, positive predictive value (PPV), and negative predictive value (NPV) were calculated from 2 × 2 contingency tables at the optimal cut-off determined by the Youden index (J). Statistical analyses were performed using R (version 4.3.1) with the pROC package.

## Supplementary Information

Supplementary Material 1.

Supplementary Material 2.

Supplementary Material 3.

## Data Availability

Endothelial RNA-seq data have been deposited in Gene Expression Omnibus (GEO) under accession number GSE302138. Nanostring data are deposited in GEO under accession number GSE303318. Mass spectrometry raw data are included as supplementary material in this manuscript.

## Abbreviations

α-SMA: Alpha-Smooth Muscle Actin
CFSE: Carboxyfluorescein succinimidyl ester
CM: Conditoned Medium
CXCL1: Chemokine C-X-C motif ligand 1
CXCR4: C-X-C chemokine receptor type 4
ESA: Early-Stage Alive lung cancer patients
ESD: Early-Stage Deceased lung cancer patients
EVPs: Extracellular vesicles and particles
HBEC: Human Bronchial Epithelial Cell
HUVEC: Human Umbilical Vein Endothelial Cells
G-MDSCs: Granulocytic myeloid-derived suppressor cells
LC: Lung cancer
MISEV: Minimal information for studies of extracellular vesicles
NK: Natural killer cells
PMNs: Pre-metastatic niches
SCR: Negative sequence control
SPARC: Secreted Protein Acidic and Rich in Cysteine
VCAM1: Vascular Cell Adhesion Molecule 1
WB: Western Blot
