# Supplementary figures and images for: Circulating extracellular vesicles from early-stage lung cancer patients trigger endothelial activation to drive pre-metastatic niche formation through synergistic miR-29a and C4A signaling
Source: J Exp Clin Cancer Res. 2026 May 13;45:151. doi: 10.1186/s13046-026-03732-4 (PMC13340091; doi:10.1186/s13046-026-03732-4)

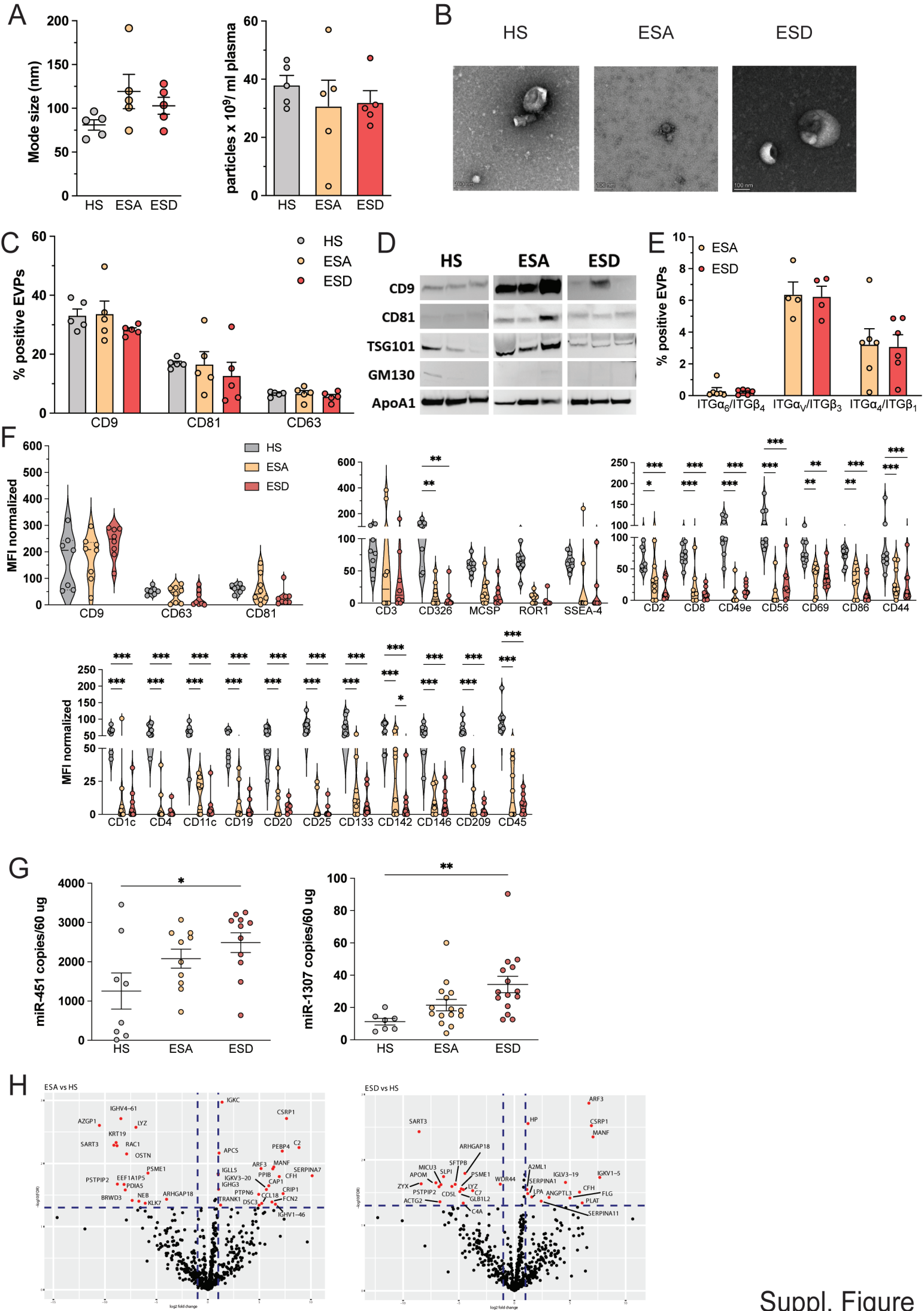

A

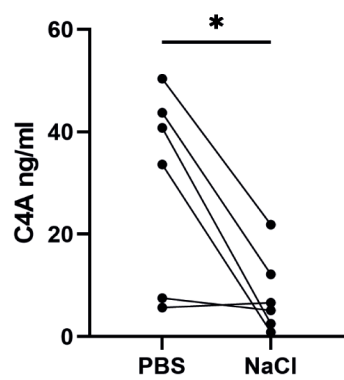

B

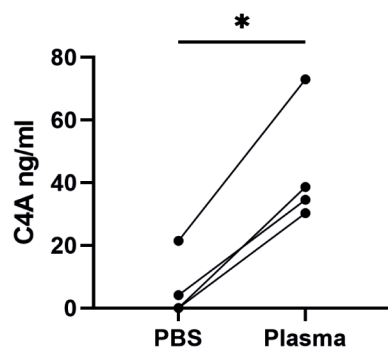

C

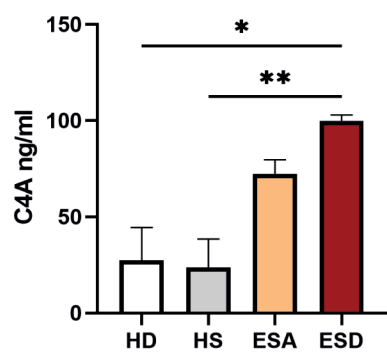

A

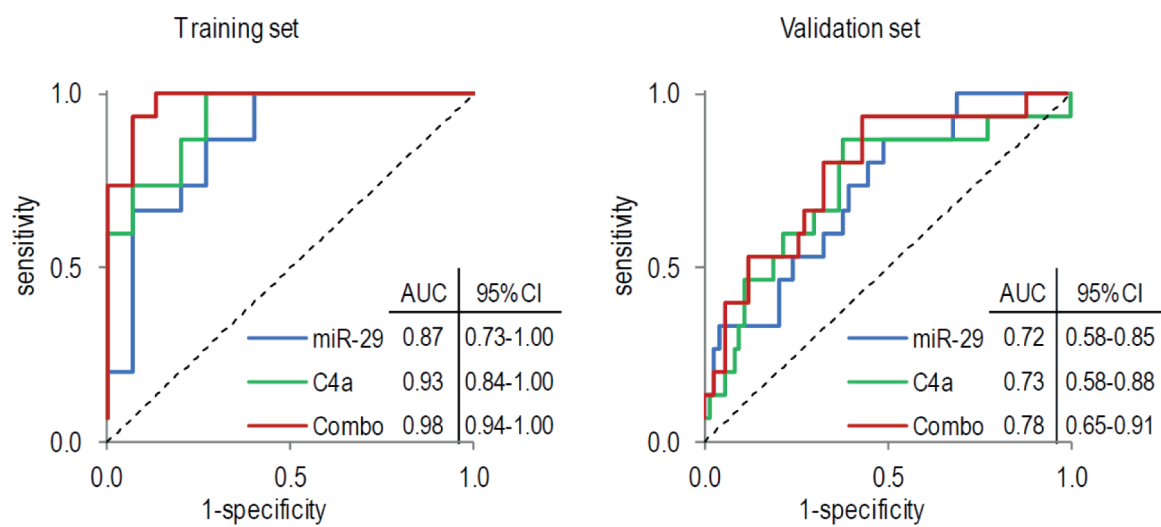

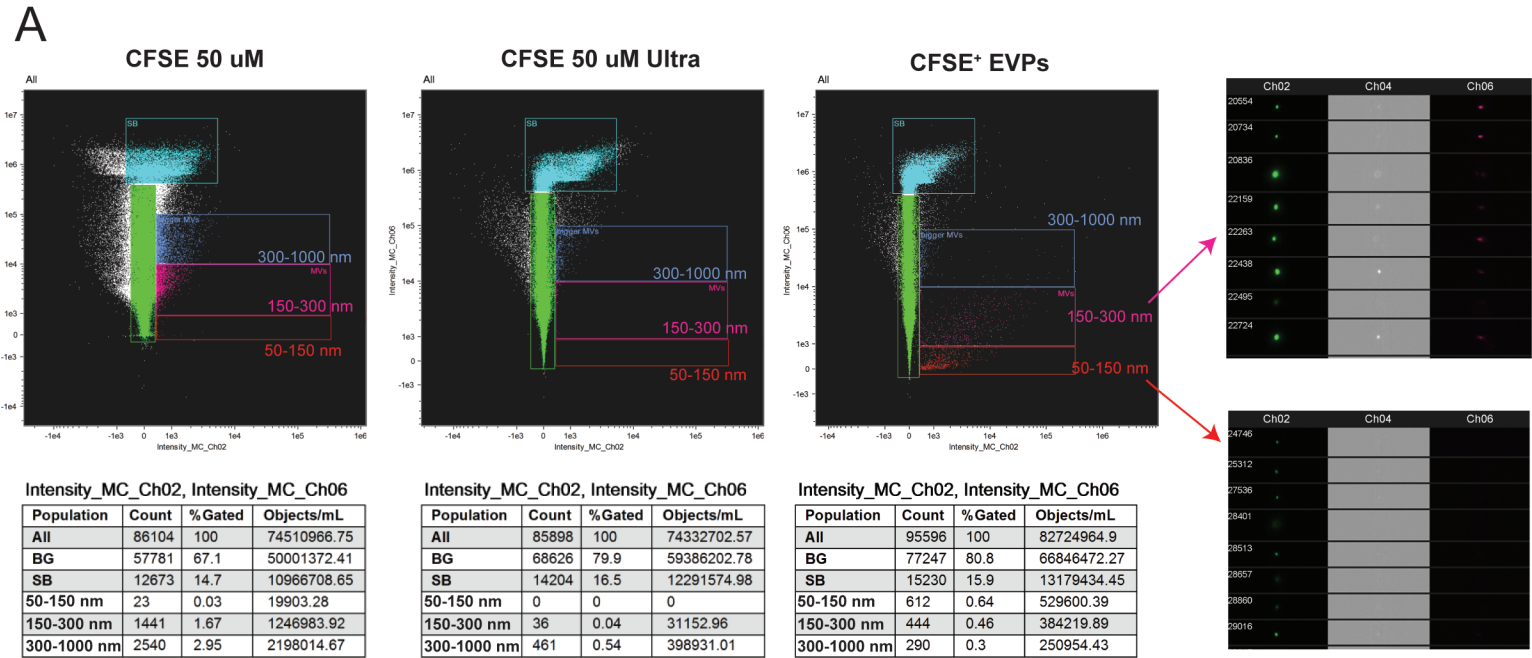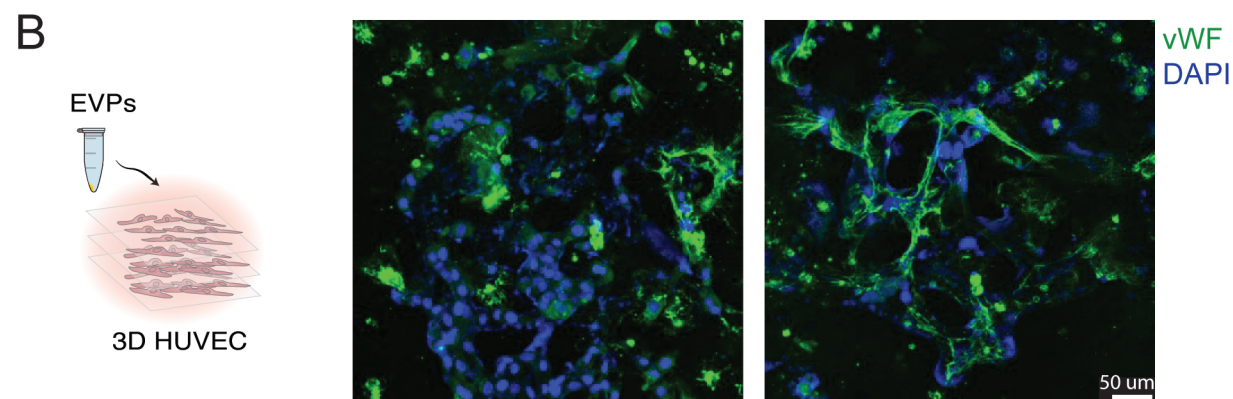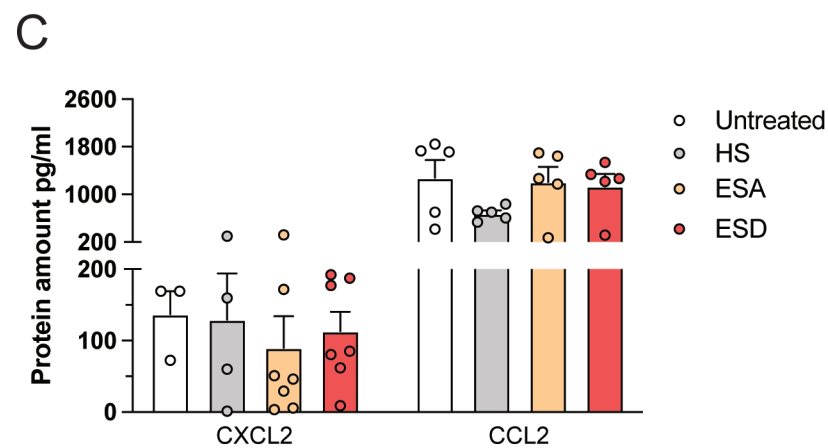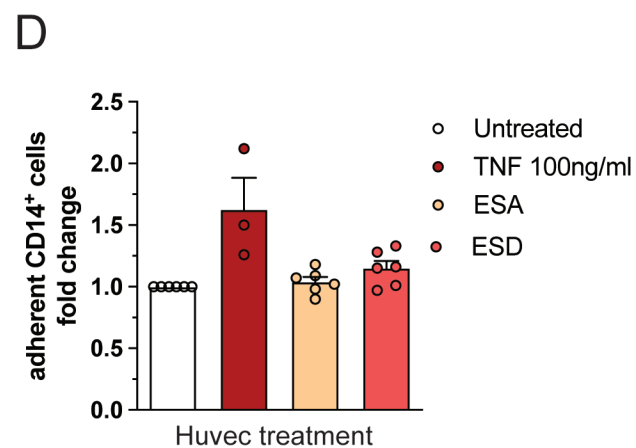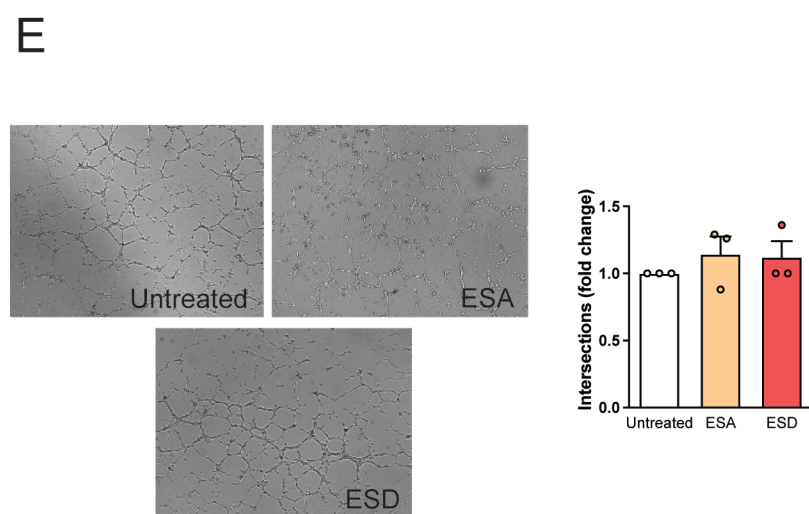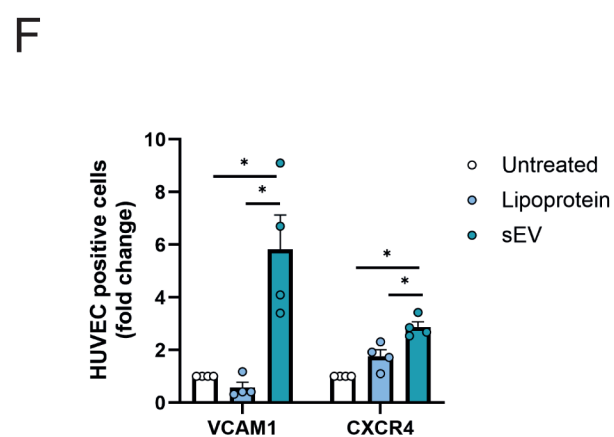

A

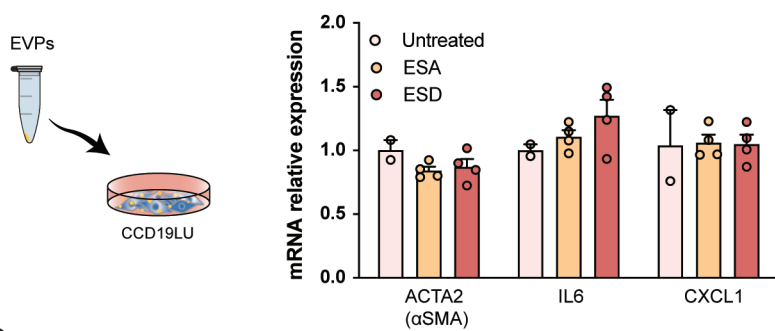

B

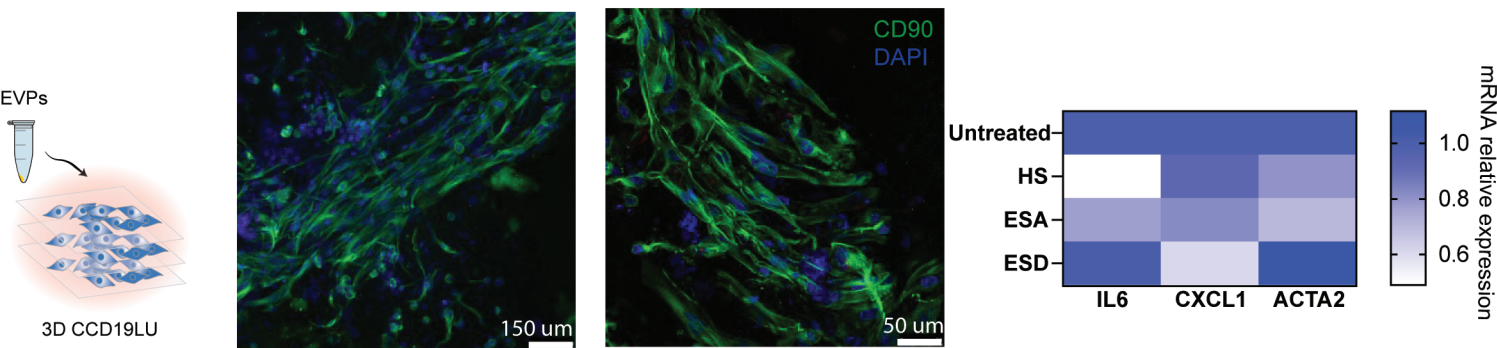

C

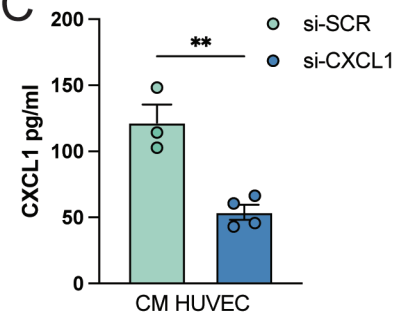

D

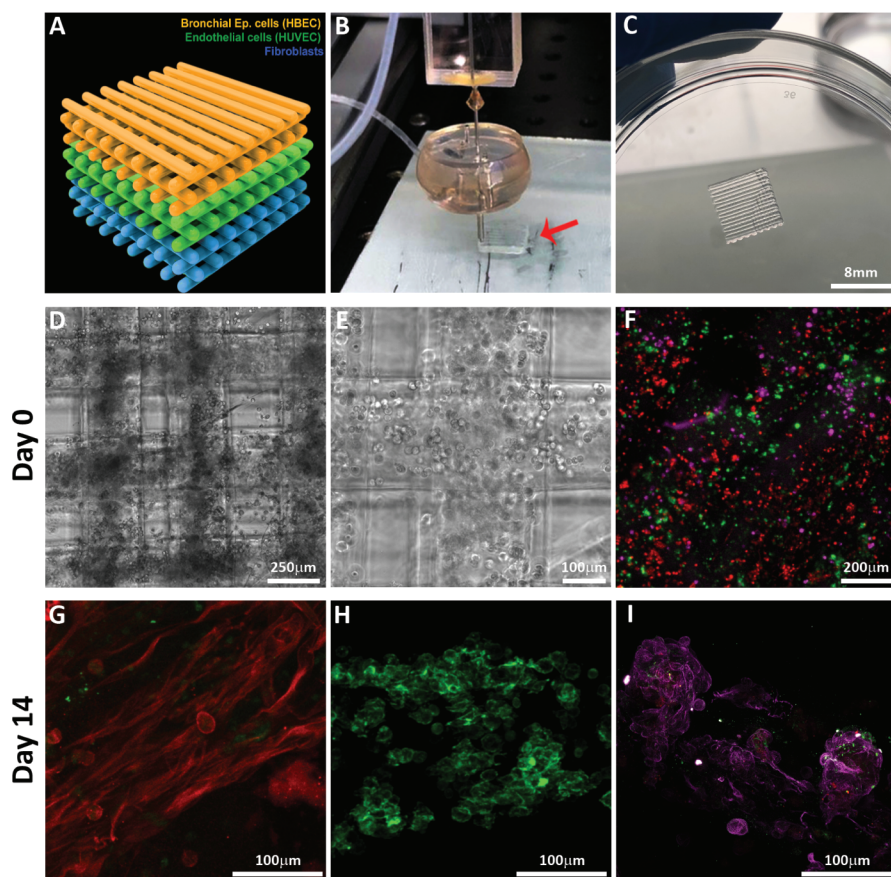

E

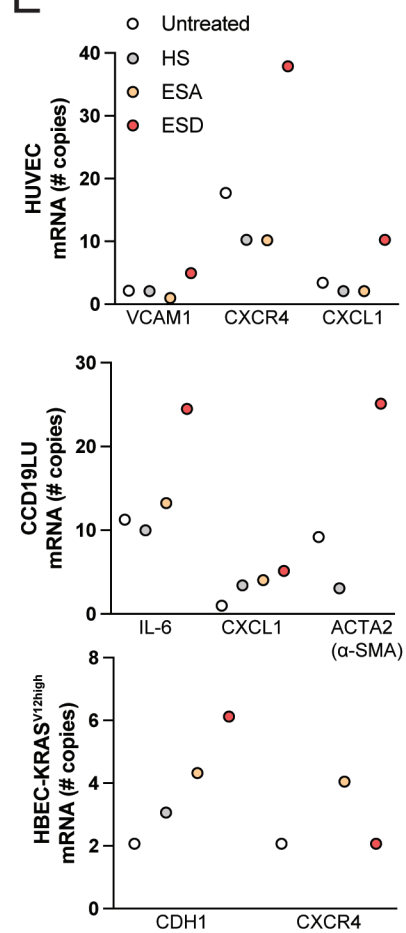

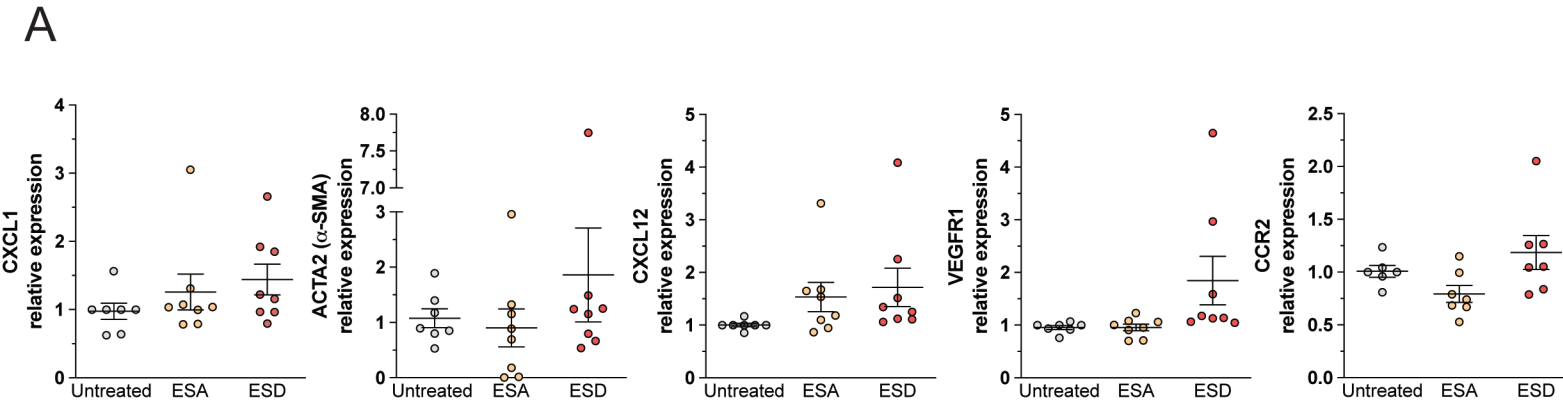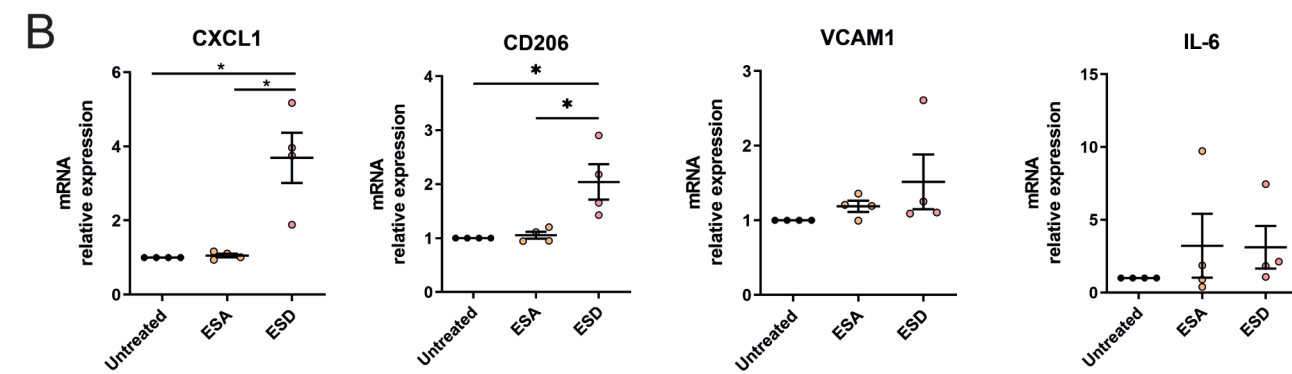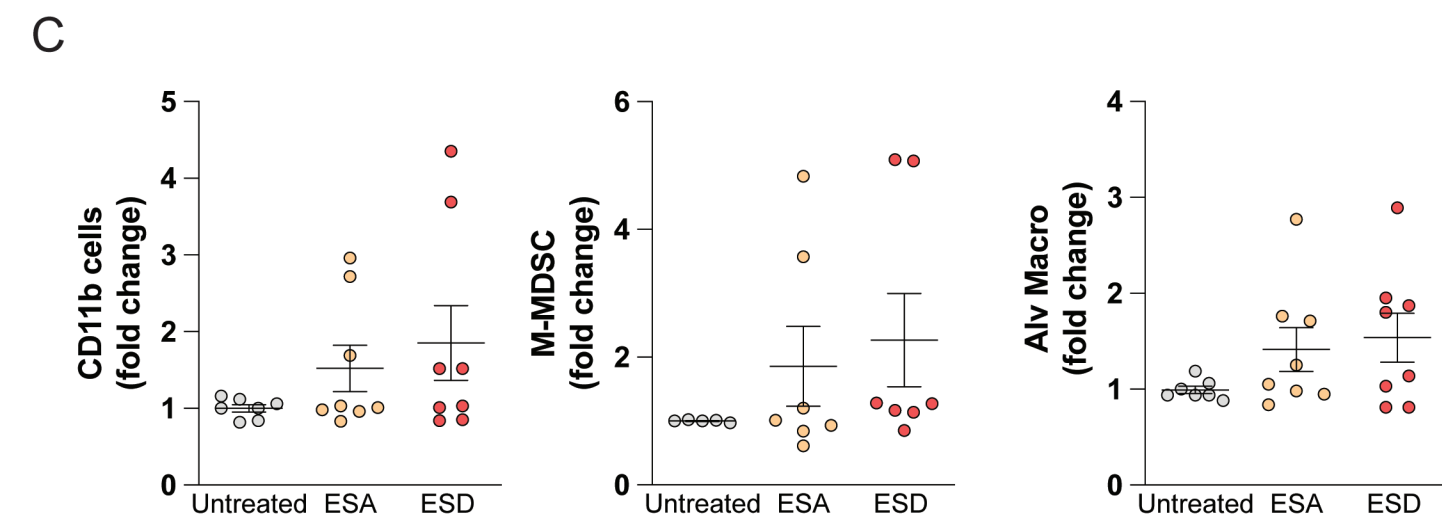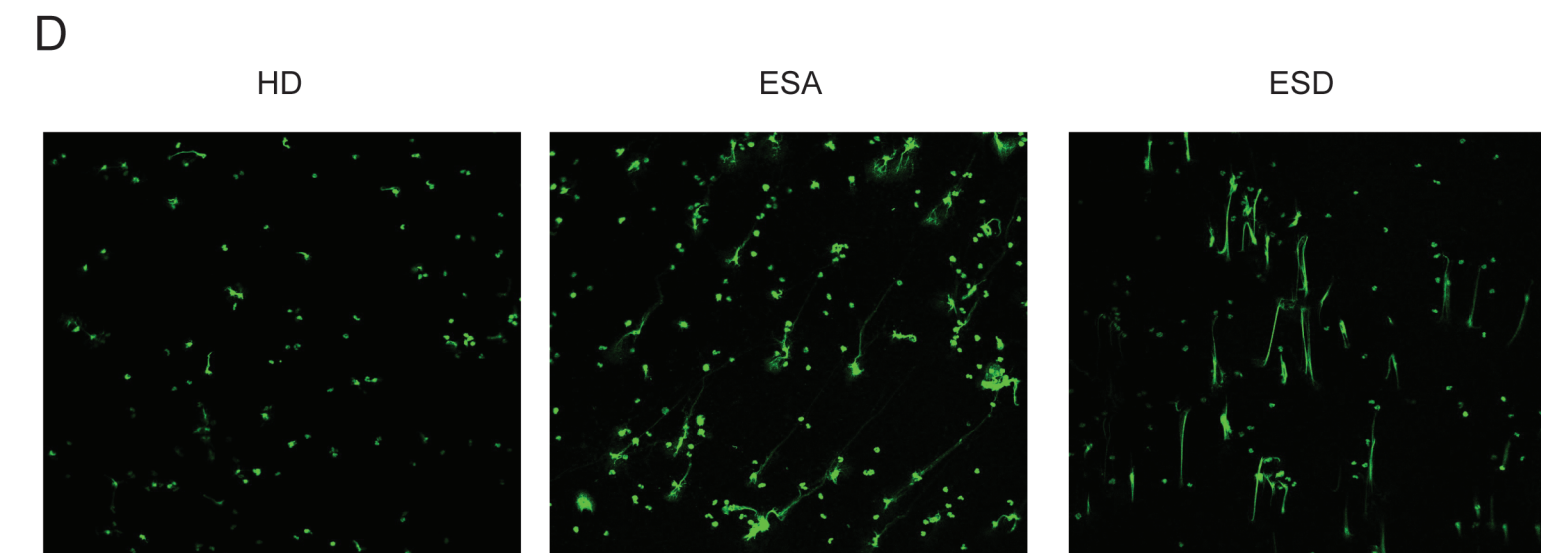

A

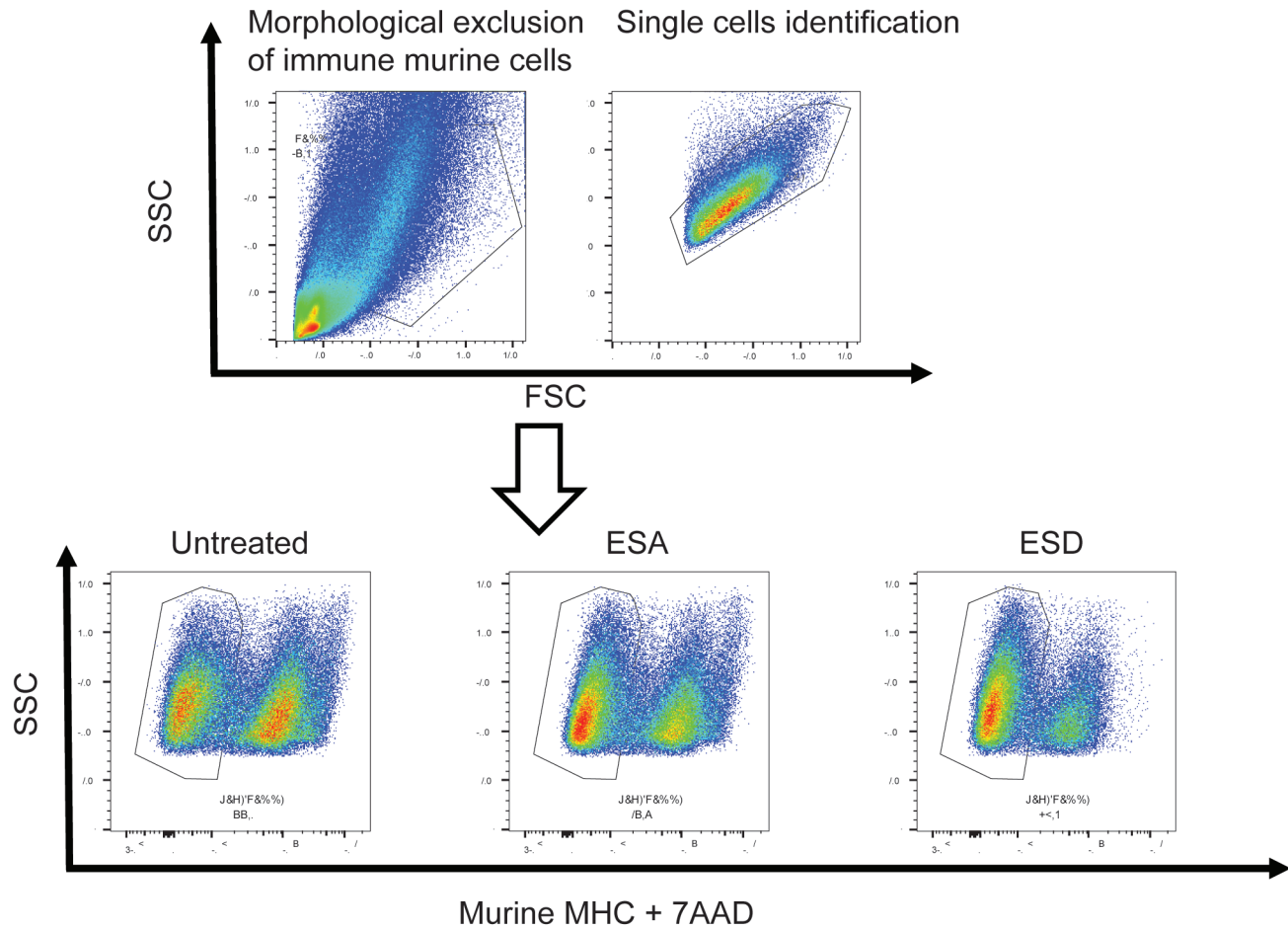

B

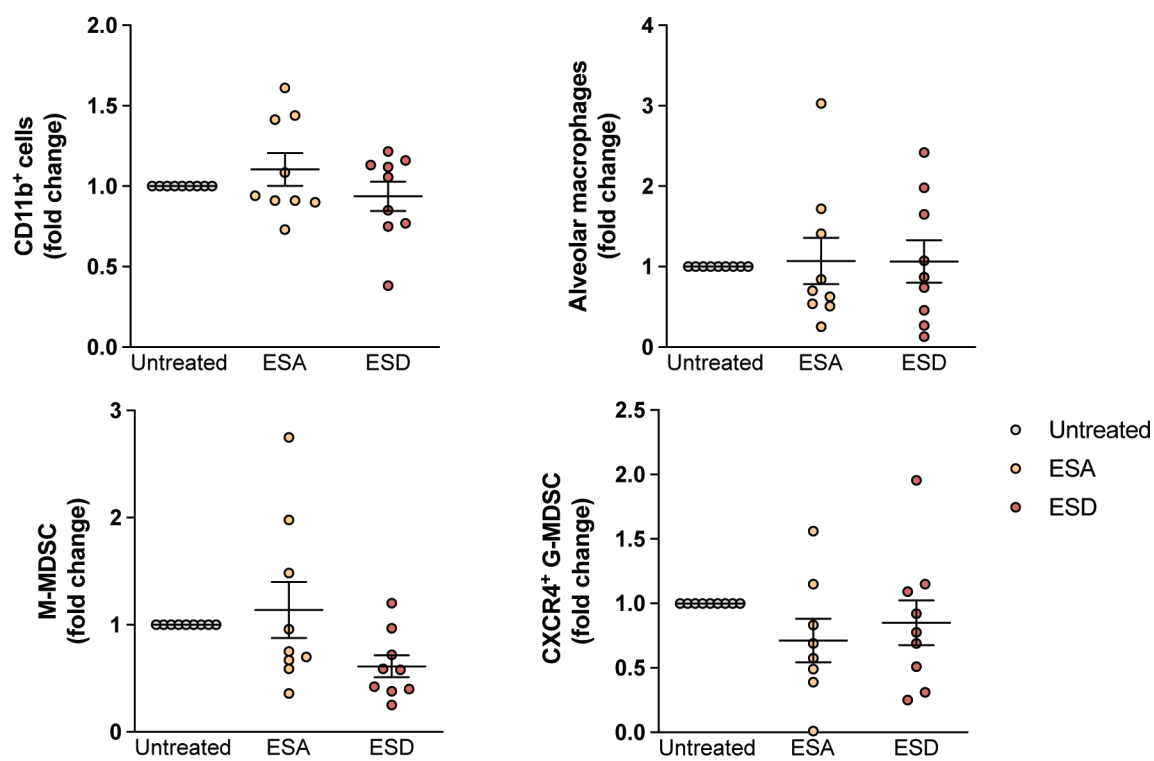

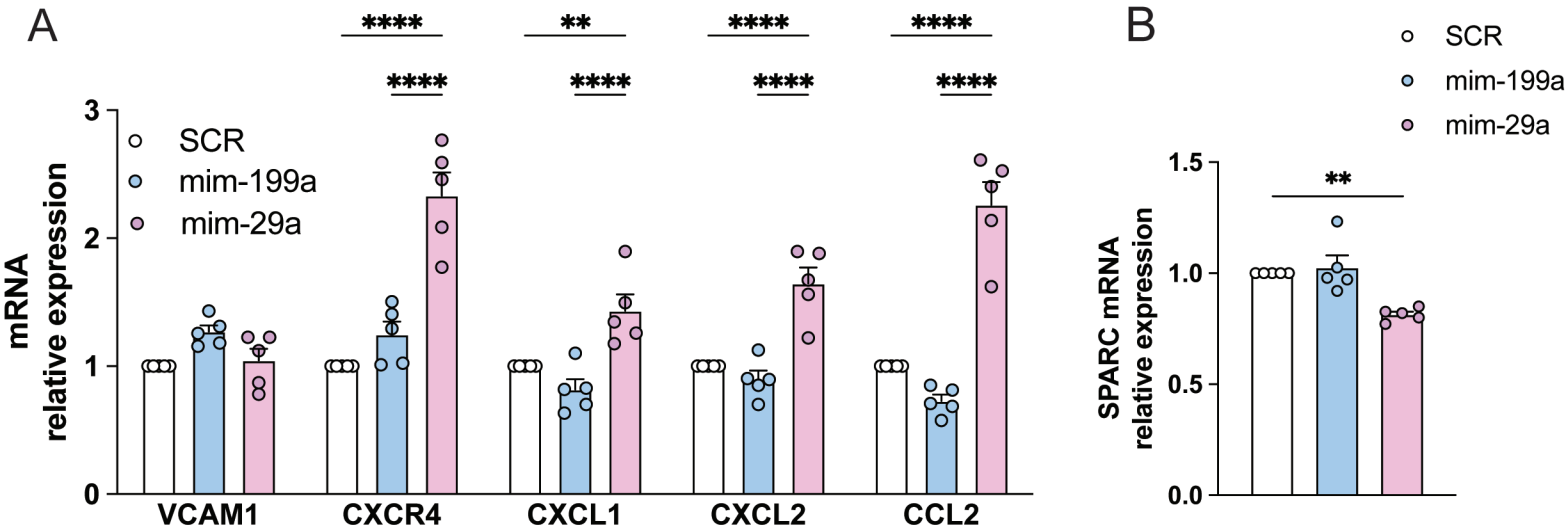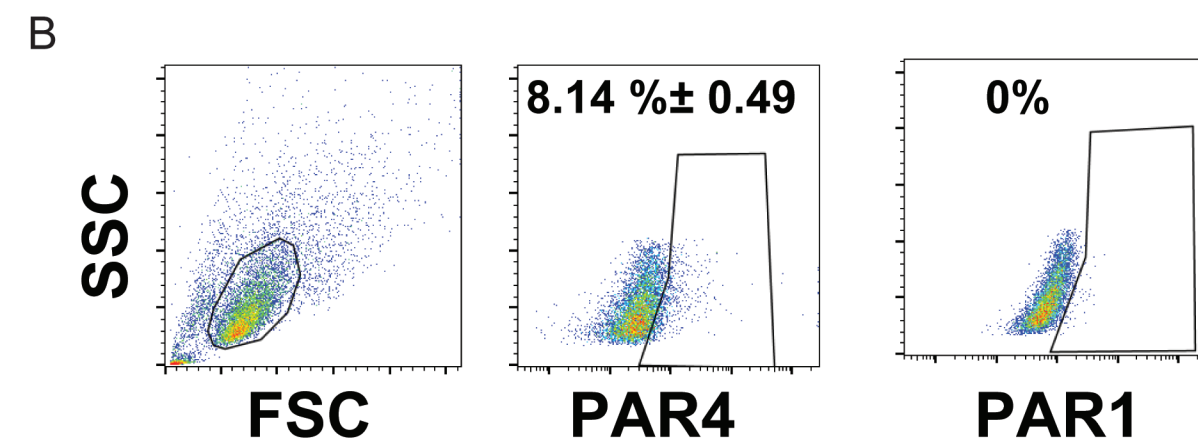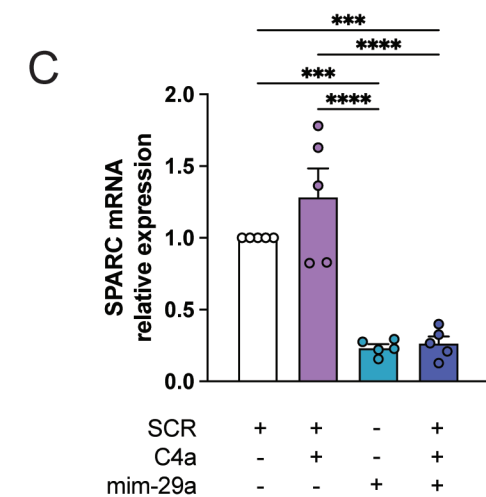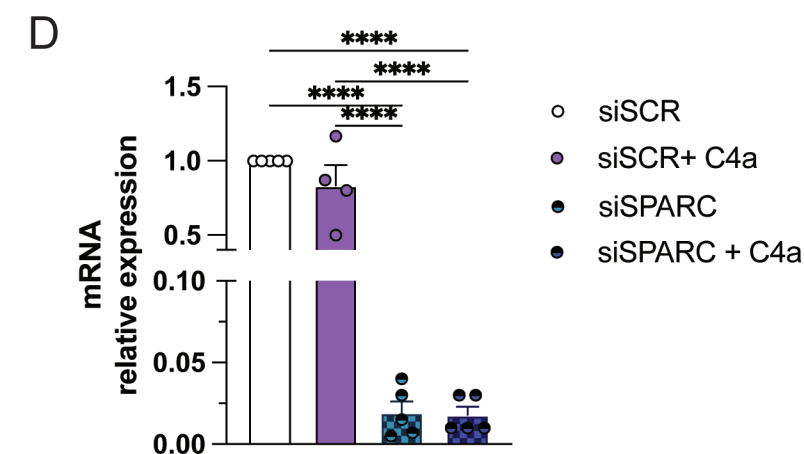

Supplement: Supplementary file 1 — Supplementary Material 1. [file 13046_2026_3732_MOESM1_ESM.pdf]
