# Supplementary material for: Circulating extracellular vesicles from early-stage lung cancer patients trigger endothelial activation to drive pre-metastatic niche formation through synergistic miR-29a and C4A signaling
Source: J Exp Clin Cancer Res. 2026 May 13;45:151. doi: 10.1186/s13046-026-03732-4 (PMC13340091; doi:10.1186/s13046-026-03732-4)
Supplement: Supplementary file 3 — Supplementary Material 3. [file 13046_2026_3732_MOESM3_ESM.docx]

**SUPPL. METHODS**

*In vitro* experiments

Sucrose Density gradient

After EV isolation via ultracentrifugation, small EV pellets were charged in a sucrose density gradient as previously reported (Radeghieri et al.). Briefly, small EVs were resuspended in 1 ml of 250 mM sucrose 10 mM Tris-HCl, pH 7.4 buffer and loaded on the top of a discontinuous sucrose gradient composed by the following sucrose solutions diluted in Tris‑HCl, pH 7.4 buffer (w/v): 15 % (600 μl), 20 % (400 μl), 25 % (400 μl), 30 % (400 μl), 40 % (400 μl), 60% (400 μl), 70% (800 µl). Then, gradients were centrifugated at 230,000 x g for 16 h at 4˚C using a SW 55 Ti swinging rotor (Beckman Coulter) without brake. Twelve fractions of 400 μl/each were recovered from the top of the gradient. After the addition of 600 μl of 0.1 μm filtered PBS, fractions were pelleted by ultracentrifugation at 100,000 x g for 2 h at 4°C using a F50L-24 X 1.5 rotor (Thermo Fisher Scientific). Eventually, fractions enriched in lipoproteins (fractions 4 and 5) and fractions enriched in small EVs (fractions 8 and 9) were resuspended in 50 μl of 0.1 μm filtered PBS and used in functional experiments.

***High-Salt washing***

To investigate the localization of C4A, ESD-EVPs were resuspended in solutions containing 1 M NaCl or PBS (as control) for 1h at 4°C as described in Försönits et al^1^. Following resuspension EVPs were pelleted and analyzed for C4A by ELISA.

***C4A adsorption***

Plasma of ESD patients were depleted from EVP using ultracentrifugation using a TLA-100.3 fixed-angle rotor at 120,000×g for 90 minutes at 4°C in TL-100 ultracentrifuge. Then, 15ug of EVP isolated from health donors were incubated at 37°C for 1h in plasma of EVP-depleted. After incubation EVPs were ultracentrifuged and analyzed using ELISA for C4A.

***ELISA for C4A determination***

The quantification of C4A fragment will be performed using commercial ELISA assays (BD Opt EIA). Briefly, 15 ug of EVPs were incubated with RIPA buffer and protease inhibitors cocktail and then added to the ELISA’s plate. The absorbance at 450 nm will be measured by the Infinite M1000 Tecan spectrophotometer microplate reader (Tecan Group Ltd).

Cell lines

Human umbilical vein endothelial cells (HUVECs) (Lonza, Basel, Switzerland) were cultured in EBM-2 supplemented with EGM-2 BulletKit (Lonza). Normal adult lung fibroblasts CCD19lu (ATCC, Manassas, VA, USA) were maintained in FBM with Kit‐Low serum (ATCC). Immortalized Human Bronchial Epithelial Cells (HBEC-KRAS^V12high^ cells, provided by Prof. J.D. Minna, UT Southwestern) were cultured in K-SFM (Thermo Fisher Scientific) supplemented with 5 ng/ml EGF and 50 μg/ml bovine pituitary extract (Thermo Fisher Scientific)^2^. All cells were maintained at 37°C in 5% CO2. Cell lines were authenticated by STR profiling and confirmed mycoplasma-negative.

*Macrophages’ isolation and differentiation*

Macrophages were differentiated from peripheral blood mononuclear cells (PBMCs) isolated using Sepmate-50 tubes (STEMCELL Technologies) from 5 ml of blood diluted 1:1 with PBS, layered over Ficoll-Paque (Sigma-Aldrich) and centrifuged at 1,200×g for 10 minutes at RT. PBMCs were collected, washed, and 1 × 10^6^ cells/ml were seeded in 12-well plates. Monocytes were selected by plastic adherence after 4 hours, then differentiated using M-CSF (20 ng/ml, Sigma-Aldrich) for 7 days.

Isolation of neutrophils and monocytes from patients’ blood

Neutrophils and monocytes were isolated from lung cancer patients blood samples following and adapting a previously published protocol by Cui et al^3^. PBMC and neutrophils were separated from 6-7 ml of blood diluted 1:1 in PBS by Ficoll-Paque. The PBMC layer present at the interphase was collected, washed and subjected to red blood cell lysis. Monocytes (CD14^+^ cells) were then isolated by magnetic activated cell sorting (MACS; Miltenyi Biotec) following the manufacturer’s instructions. CD14^+^ cells were isolated automatically using a autoMACS Pro Separator and the Possel program.

Neutrophil’s isolation was performed on the cell pellet present at the bottom of the tube after Ficoll separation. Red blood cells were then removed until the pellet appeared clear from erythrocytes.

Extracellular traps formation was evaluated treating normal density neutrophils (2x104) seeded onto poly-D-Lysine coated glasses with 10 ug of EVs for 4h and evaluating the incorporation of the DNA dye SITOX green through confocal microscopy.

Functional Assays

*Matrigel tube formation*

EVP-treated HUVECs (2×10^4^) were seeded on Matrigel-coated 8-well chamber slides (Thermo Fisher, RS Glass) with Corning Matrigel LDEV-free (Euroclone). After 5 hours at 37°C, structures were fixed with 2% PFA and quantified by counting intersection points at 20X magnification in five random fields.

Adhesion experiments

HUVECs (7 × 10^5^ cells/well) were seeded in 6-well plates and treated with EVPs for 48 h or TNF-α (100 ng/ml) for 4 hours before the addition of PKH26^+^ immune cells. Labelled immune cells were then added at the following densities: 1 × 10⁶ cells/well for neutrophils, 2 × 10⁵ cells/well for CD14^+^ cells. The control wells included both untreated endothelial cells alone and labelled cells alone. The co-cultures were incubated for 2 hours to facilitate cell interaction and adhesion. Subsequently, the wells were washed three times with PBS using a rocking platform shaker to remove any unbound cells. Adherent cells were harvested, centrifuged and analyzed by flow cytometry using FlowJo software (TreeStar, Ashland, OR, USA). A minimum of 5 × 10^4^ events were acquired for each tube and the number of PKH26^+^ cells was used for analysis.

Migration experiments

Neutrophils were obtained as described above and seeded at a concentration of 1 × 10^5^ cells/well into the top chamber of 24-well cell culture inserts with an 8 µm pore size (Corning, Glandale). In the lower chamber, 750 µL of CM from treated and untreated HUVECs, serum free medium (negative control) or serum free medium + CXCL1 (100 ng/ml) (positive control) were added. After 5 hours of incubation, migrated cells were harvested from the lower chamber and the number of migrated cells was evaluated by cell counting.

Flow cytometry

*EV tetraspanin and integrin profile analysis*

Flow cytometry was performed using standard protocols on FACSCanto II and LSRFortessa instruments (BD Biosciences). 30 μg EVP were analyzed after coupling to aldehyde/sulfate latex beads (4 μm, Invitrogen) and stained with tetraspanin antibodies (anti-CD9, anti-CD81, Cell Signaling; anti-CD63, Abcam), secondary antibodies Alexa Fluor 647-conjugated goat anti-rabbit IgG (Thermo Fisher Scientific) or DyLight 488-conjugated goat anti-mouse IgG (Bethyl) were used for tetraspanin characterization. Fluorochrome-conjugated antibody listed in **Suppl. Table** **S10** were used for integrin profiling. The origin of plasma EVs was determined using MACSPlex EV Kit (Miltenyi Biotec). EVs (15 μg/sample) were analyzed in accordance with the manufacturer’s instructions. A minimum of 10000 events were recorded for each sample and analysis was performed using mean fluorescence values (subtracted from background values, using "blank" samples) and normalized to the mean signal intensity of the three tetraspanin CD9/63/81. Negative values were excluded from the graph. Data analysis was performed using FlowJo software.

*Cellular staining*

Cellular analyses used fluorochrome-conjugated antibodies with 15-30 min incubations at 4°C or RT. For HUVEC the following antibodies were used: CD34 (APC-Cy7), CXCR4 (PE-Cy7), and VCAM1-BV421 (all BioLegend, San Diego, CA, USA). For *ex vivo* tissue analysis, mouse lungs were mechanically and enzymatically digested using the Tumor Dissociation Kit, Miltenyi Biotec and the gentleMACS™ Dissociator. After red blood cell removal, the cells were stained with the antibodies listed in **Suppl. Table** **S11**. Samples were analyzed using LSRFortessa (BD Bioscience. For the colonization assay, lung tumor cells were identified in lung-dissociated tissues by Flow Cytometry as 7-aminoactinomycin D^−^ (7-AAD, BioLegend)/mouse major histocompatibility complex (MHC, Invitrogen) class I^−^ cells as already described ^4^.

*Imagestream acquisition and analysis*

HUVEC and EVPs were imaged using ImagestreamX MarkII System (Amnis, part of Cytek® Biosciences), a multispectral imaging flow cytometer that combines the information content coming from microscopy with the statistical power typical of flow cytometry.

The instrument is equipped with 3 lasers (405nm, 488nm and 642nm), 6-channels CCD camera and Multimag option. All samples were run with the same acquisition settings: 15 µg of EVs stained with CFSE were diluted 1:100 and imaged with the 60X_0.9NA objective at low speed and high sensitivity, core size 7 μm and the “Remove Beads” option unchecked in order to detect microparticles but also speed beads running throughout the acquisition. Excitation lasers were all run to maximal power, which means 200mW for the 488nm laser and 70mW for the 785nm laser. EVPs were introduced into the instrument and, as soon as the acquisition started, the events were collected for 3 minutes. Samples without EVs containing only CFSE dye + PBS were processed as well as the EVPs samples (ultracentrifuged) and used as negative controls.

Cells were imaged with the same acquisition settings used for the EVs, except for the 785nm laser that was set at 0.9mW and for the Remove Beads option that was checked. At least 10.000 events were collected for each sample.

Images were analyzed using IDEAS 6.2 software as described below.

- To confirm the presence of EVPs in the sample, Intensity_Ch02 (CFSE) was plotted against Intensity_Ch06 (side scatter) and EVs were identified as events with very low scattered light and low/mid green fluorescence intensity. EVPs were easily distinguished from speed beads which were instead characterized by very high scatter intensity and no fluorescent signal.

To avoid false positive events, both CFSE dye alone diluted at the same concentration used to stain EVPs were ran on the system to ensure no events were detected.

- HUVEC were first gated for focused events using Gradient root mean square feature (RMS) on the BF image and then single cells were identified using area vs aspect ratio feature. To visualize the EVs internalization by endothelial cells, first Intensity feature vs Max pixel intensity feature was used to identify cells that were positive for green spotted signal. Then, since EVs are detected as green spots inside the cells, the Internalization feature applied on an eroded (4 pixels) brightfield mask was used identify cells with green spots in the cytoplasm.

***RNA Extraction***

Total RNA was extracted from EVPs (60 μg) and cell pellets using Maxwell RSC Instrument with Maxwell RSC miRNA Tissue Kit (Promega) according to manufacturer's instructions. RNA quantification and quality were assessed using NanoDrop spectrophotometer.

*Reverse transcription*

***mRNA Analysis***: cDNA was synthesized from 250-500 ng total RNA using 1 μl random primers (0.5 μg/μl, Invitrogen), 1 μl dNTP (10 mM, Invitrogen), nuclease-free water to reach 12 μl of total volume and then incubated at 70°C for 10 minutes. The reaction mix (4 μl 5X First-Strand Buffer, 2 μl 0.1 M DTT, 1 μl RNaseOUT™, 1 μl SuperScript™ III RT; Invitrogen) was then added and samples were incubated for 60 minutes at 42°C. The reaction was terminated at 70°C for 15 minutes.

***miRNA Analysis***: Reverse transcription was performed using TaqMan® microRNA RT kit with custom multiplex primer pools. RT primers (25 μl each 5X, 250 nM) for hsa-miR-199a-3p, hsa-miR-29a-3p, hsa-miR-451a, hsa-miR-1307 were pooled, dried and resuspended to create 5X multiplex pool (62.5 nM each). RNA (20 ng) was reverse transcribed using this pool with standard TaqMan conditions.

Digital PCR

Digital PCR was performed as previously described by Conte et al ^5^ using QuantStudio 3D system (Thermo Fisher) with TaqMan assays. Samples were loaded onto chips using automated loader, amplified (40 cycles: 2 min at 56°C, 30 s at 98°C) and hsa-miR-199a-3p, hsa-miR-29a-3p, hsa-miR-451a, hsa-miR-1307 number of copies were analyzed with QuantStudio 3D software using 10,000 data point threshold for quality control.

Quantitative Real-Time PCR

Quantitative real-time-polymerase chain reaction (qPCR) was conducted using Taqman Universal Master Mix II (Thermo Fisher Scientific) according to the manufacturer's guidelines. Briefly, amplification reactions were performed using MicroAmp® Fast Optical 96-Well Reaction Plates (Thermo Fisher Scientific) and 20 μl of the reaction mix was added to each well (containing 5ng of cDNA and 2ul of primers). Two technical replicates were performed for each sample. QuantStudio™ 12 K Flex Real-Time PCR System (Thermo Fisher Scientific) and its associated software (Thermo Fisher Scientific) were used for the reaction and analysis. Cycling conditions: 95°C for 10 minutes, then 40 cycles of 95°C for 15 seconds and 60°C for 60 seconds. β2M served as endogenous control; data were analyzed using the 2^-ΔΔCt method.

Immunochemical assays

*Immunofluorescence*

The 3D bioprinted constructs were washed, fixed with PFA 4% for 2 hours and incubated in a blocking solution containing 5% BSA and 0.1% Triton X for 1 hour. Primary antibodies against CD90 (Abcam) and sheep anti-von Willebrand (vWF; Abcam) were diluted 1:100 in 0,5% BSA) and incubated overnight at 4°C. Secondary antibodies FITC or TRITC conjugated (Jackson Immunoresearch) diluted 1:200 were incubated for 1h. After washing, the slides were mounted with Vectashield (Thermo Fisher Scientific) and DAPI. The images were acquired using laser scanning confocal microscopy (Leica Microsystem).

*Evaluation of cytokine and chemokine presence in endothelial CM by ELISA*

To investigate cytokine production by treated endothelial cells, 1 ml of EVP-treated endothelial cells CM was analyzed by Proteome Profiler Human Cytokine Array Kit (R&D Systems) according to the manufacturer’s instructions. Images were acquired using a MINI HD9 Western Blot Imaging System (Cleaver Scientific Ltd., UK).

The amounts of CXCL1, CXCL2 and CCL2 were evaluated using a Quantikine ELISA Kit (R&D Systems) specific for each cito/chemokine, in accordance with the manufacturer’s instructions. The concentration of cytokines present in the CM was determined using regression analysis.

Ex vivo IHC staining and analysis

IHC analysis was performed on formalin-fixed, paraffin-embedded (FFPE) lung tissue sections to detect pan-cytokeratin (CK AE1 AE3, Dako-Agilent). The acquired images were then analyzed using ImageJ software and ImageJ plugin IHC Profiler. A total of 8-10 fields per treatment group were assessed to ensure the collection of accurate and representative data.

**Suppl. Table S1. List of miRNAs upregulated in ESD vs ESA-EVPs**

|  | **microRNAs** | **P-value** |
| --- | --- | --- |
| 1 | miR-1307-3p | 0.0057 |
| 2 | miR-451a | 0.0171 |
| 3 | miR-29a-3p | 0.0177 |
| 4 | miR-199a-3p | 0.0258 |
| 5 | miR-548ah-5p | 0.0700 |
| 6 | miR-514b-5p | 0.0854 |
| 7 | miR-608 | 0.0869 |
| 8 | miR-802 | 0.0893 |

**Suppl. Table S2. List of proteins modulated in ESD and ESA-EVPs**

| **ESD vs ESA** | **logFC** | **p-value** |
| --- | --- | --- |
| **SERPINA7** | -10.844 | 0.0080 |
| **ORM2** | 3.962 | 0.0111 |
| **PGLYRP2** | 3.777 | 0.0118 |
| **RAC1** | 6.336 | 0.0125 |
| **SFTPB** | -4.925 | 0.0128 |
| **C4A** | -5.126 | 0.0140 |
| **KRT77** | -4.925 | 0.0197 |
| **CSTA** | -5.165 | 0.0205 |
| **EEF1G** | -5.820 | 0.0246 |
| **AZGP1** | 5.698 | 0.0299 |
| **CAP1** | -4.447 | 0.0313 |
| **PIP4K2A** | 3.315 | 0.0378 |
| **MMP14** | 6.187 | 0.0383 |
| **DCD** | -4.631 | 0.0399 |
| **FGL1** | 8.029 | 0.0472 |
| **COL6A3** | -4.552 | 0.0478 |

**Suppl. Table S3. List of proteins modulated in ESD and HS-EVPs**

| **ESD vs HS** | **logFC** | **p-value** |
| --- | --- | --- |
| **HP** | 1.151 | 0.001 |
| **ARF3** | 6.591 | 0.002 |
| **CSRP1** | 6.922 | 0.003 |
| **IGLL5** | 0.874 | 0.010 |
| **A2ML1** | 1.086 | 0.011 |
| **IGKC** | 0.801 | 0.012 |
| **C7** | -4.131 | 0.016 |
| **IGKV1-5** | 7.785 | 0.017 |
| **ARHGAP18** | -4.337 | 0.018 |
| **SLPI** | -6.484 | 0.018 |
| **IGLV3-19** | 4.633 | 0.018 |
| **APOM** | -7.051 | 0.024 |
| **SFTPB** | -5.248 | 0.024 |
| **ALB** | 0.755 | 0.026 |
| **CD5L** | -5.696 | 0.026 |
| **ZYX** | -8.204 | 0.026 |
| **PSTPIP2** | -6.746 | 0.027 |
| **LPA** | 1.483 | 0.027 |
| **IGFBP4** | 0.616 | 0.028 |
| **LYZ** | -4.580 | 0.030 |
| **SERPINA1** | 1.124 | 0.031 |
| **CFH** | 5.944 | 0.032 |
| **APCS** | 0.752 | 0.033 |
| **FLG** | 5.127 | 0.034 |
| **ANGPTL3** | 3.124 | 0.040 |
| **F5** | 0.947 | 0.041 |
| **C4A** | -4.760 | 0.048 |
| **KLK7** | -5.628 | 0.048 |

**Suppl. Table S4. List of proteins modulated in ESA and HS-EVPs**

| **ESA vs HS** | **logFC** | **p-value** |
| --- | --- | --- |
| **IGKC** | 1.319 | 0.000 |
| **CSRP1** | 7.598 | 0.002 |
| **AZGP1** | -10.457 | 0.003 |
| **LYZ** | -6.965 | 0.003 |
| **APCS** | 1.084 | 0.005 |
| **HP** | 0.962 | 0.005 |
| **RAC1** | -8.705 | 0.006 |
| **IGLL5** | 0.986 | 0.006 |
| **IGHG3** | 0.951 | 0.011 |
| **MSN** | -0.667 | 0.011 |
| **PPIB** | 6.416 | 0.011 |
| **SERPINA7** | 10.070 | 0.012 |
| **ARF3** | 5.087 | 0.013 |
| **CFH** | 7.052 | 0.016 |
| **CFHR3** | 0.661 | 0.019 |
| **APOA5** | 0.742 | 0.021 |
| **CAP1** | 5.961 | 0.021 |
| **PSTPIP2** | -8.650 | 0.023 |
| **IGKV3-20** | 5.780 | 0.023 |
| **CRIP1** | 7.470 | 0.026 |
| **ADH1C** | 2.427 | 0.029 |
| **ALB** | 0.759 | 0.031 |
| **PDIA5** | -7.714 | 0.031 |
| **PTPN6** | 4.893 | 0.032 |
| **FCN2** | 6.464 | 0.036 |
| **OLA1** | -0.603 | 0.036 |
| **TLN1** | -0.914 | 0.040 |
| **ARHGAP18** | -3.906 | 0.040 |
| **IGHG1** | 0.837 | 0.041 |
| **CFI** | 0.669 | 0.041 |
| **KLK7** | -6.007 | 0.044 |
| **FLG** | 5.013 | 0.045 |
| **C7** | -3.463 | 0.047 |

**Suppl. Table S5. List of pathways involved in ESD-treated HUVECs**

| **ID** | **Description** | **GeneRatio** | **p-value** | **p-adjusted** |
| --- | --- | --- | --- | --- |
| GO:0045765 | regulation of angiogenesis | 24/263 | 0.0000000002 | 0.0000005 |
| GO:1901342 | regulation of vasculature development | 24/263 | 0.0000000002 | 0.0000005 |
| GO:0001667 | ameboidal-type cell migration | 28/263 | 0.0000000005 | 0.0000006 |
| GO:0043542 | endothelial cell migration | 21/263 | 0.0000000007 | 0.0000006 |
| GO:0050673 | epithelial cell proliferation | 27/263 | 0.0000000012 | 0.0000009 |
| GO:0072001 | renal system development | 22/263 | 0.0000000014 | 0.0000009 |
| GO:0010631 | epithelial cell migration | 23/263 | 0.0000000034 | 0.0000017 |
| GO:0001935 | endothelial cell proliferation | 17/263 | 0.0000000046 | 0.0000017 |
| GO:0090130 | tissue migration | 23/263 | 0.0000000051 | 0.0000017 |
| GO:0001822 | kidney development | 20/263 | 0.0000000236 | 0.0000073 |
| GO:0003158 | endothelium development | 13/263 | 0.0000001261 | 0.0000335 |
| GO:0061448 | connective tissue development | 18/263 | 0.0000001449 | 0.0000360 |
| GO:0048863 | stem cell differentiation | 16/263 | 0.0000004937 | 0.00010217 |
| GO:0001569 | branching involved in blood vessel morphogenesis | 7/263 | 0.0000006149 | 0.00012001 |
| GO:0040013 | negative regulation of locomotion | 21/263 | 0.0000006443 | 0.00012001 |
| GO:0031589 | cell-substrate adhesion | 19/263 | 0.0000007856 | 0.00013936 |
| GO:0006935 | chemotaxis | 22/263 | 0.0000008711 | 0.00014749 |
| GO:0042330 | taxis | 22/263 | 0.0000009354 | 0.00015149 |
| GO:0090200 | positive regulation of release of cytochrome c from mitochondria | 6/263 | 0.0000010689 | 0.00016516 |
| GO:0001763 | morphogenesis of a branching structure | 14/263 | 0.0000012326 | 0.00017659 |
| GO:0030336 | negative regulation of cell migration | 19/263 | 0.0000015161 | 0.00020916 |
| GO:0051216 | cartilage development | 14/263 | 0.0000016459 | 0.00021896 |
| GO:2000146 | negative regulation of cell motility | 19/263 | 0.0000027145 | 0.00033773 |
| GO:0071900 | regulation of protein serine/threonine kinase activity | 16/263 | 0.0000032317 | 0.00038832 |
| GO:0030323 | respiratory tube development | 13/263 | 0.0000036417 | 0.00042391 |
| GO:0060326 | cell chemotaxis | 17/263 | 0.0000037936 | 0.00042821 |
| GO:0010633 | negative regulation of epithelial cell migration | 10/263 | 0.0000059130 | 0.00064782 |
| GO:0050680 | negative regulation of epithelial cell proliferation | 12/263 | 0.0000067558 | 0.00071901 |
| GO:0010596 | negative regulation of endothelial cell migration | 9/263 | 0.0000112819 | 0.00113581 |
| GO:0060541 | respiratory system development | 13/263 | 0.0000118350 | 0.00116015 |
| GO:0009612 | response to mechanical stimulus | 13/263 | 0.0000124402 | 0.0011839 |
| GO:0050900 | leukocyte migration | 18/263 | 0.0000133479 | 0.0011839 |
| GO:0045766 | positive regulation of angiogenesis | 12/263 | 0.0000133487 | 0.0011839 |
| GO:1904018 | positive regulation of vasculature development | 12/263 | 0.0000133487 | 0.0011839 |
| GO:0071560 | cellular response to transforming growth factor beta stimulus | 15/263 | 0.0000148099 | 0.00128295 |
| GO:0030324 | lung development | 12/263 | 0.0000156896 | 0.00132827 |
| GO:0002040 | sprouting angiogenesis | 12/263 | 0.0000174426 | 0.00141247 |
| GO:1903131 | mononuclear cell differentiation | 20/263 | 0.0000180890 | 0.00143365 |
| GO:0060562 | epithelial tube morphogenesis | 16/263 | 0.0000253237 | 0.00192512 |
| GO:1903706 | regulation of hemopoiesis | 18/263 | 0.0000293442 | 0.00218614 |
| GO:0030217 | T cell differentiation | 15/263 | 0.0000338022 | 0.00246888 |
| GO:0043405 | regulation of MAP kinase activity | 10/263 | 0.0000349224 | 0.00250166 |
| GO:0044344 | cellular response to fibroblast growth factor stimulus | 9/263 | 0.0000376639 | 0.00264713 |
| GO:0090199 | regulation of release of cytochrome c from mitochondria | 6/263 | 0.0000387870 | 0.00267559 |
| GO:0007588 | excretion | 5/263 | 0.0000490173 | 0.00331981 |
| GO:0032835 | glomerulus development | 7/263 | 0.0000534156 | 0.00355309 |
| GO:0050729 | positive regulation of inflammatory response | 10/263 | 0.0000623106 | 0.0040345 |
| GO:0048066 | developmental pigmentation | 6/263 | 0.0000635169 | 0.0040345 |
| GO:0071774 | response to fibroblast growth factor | 9/263 | 0.0000639022 | 0.0040345 |
| GO:0043535 | regulation of blood vessel endothelial cell migration | 10/263 | 0.0000658512 | 0.00408826 |
| GO:0045446 | endothelial cell differentiation | 9/263 | 0.0000680647 | 0.00415641 |
| GO:0043491 | phosphatidylinositol 3-kinase/protein kinase B signal transduction | 14/263 | 0.0000702450 | 0.00422037 |
| GO:0010810 | regulation of cell-substrate adhesion | 12/263 | 0.0000732503 | 0.00428557 |
| GO:0003014 | renal system process | 9/263 | 0.0000770770 | 0.00428557 |
| GO:2000027 | regulation of animal organ morphogenesis | 9/263 | 0.0000770770 | 0.00428557 |
| GO:0048754 | branching morphogenesis of an epithelial tube | 10/263 | 0.0000775176 | 0.00428557 |
| GO:0072006 | nephron development | 10/263 | 0.0000775176 | 0.00428557 |
| GO:0003159 | morphogenesis of an endothelium | 4/263 | 0.0000805342 | 0.00428557 |
| GO:0061154 | endothelial tube morphogenesis | 4/263 | 0.0000805342 | 0.00428557 |
| GO:0034446 | substrate adhesion-dependent cell spreading | 8/263 | 0.0000872373 | 0.00457688 |
| GO:0010822 | positive regulation of mitochondrion organization | 7/263 | 0.0000914423 | 0.00473087 |
| GO:0019934 | cGMP-mediated signaling | 5/263 | 0.0000935421 | 0.00477321 |
| GO:0097306 | cellular response to alcohol | 8/263 | 0.000100341 | 0.00505093 |
| GO:0001937 | negative regulation of endothelial cell proliferation | 7/263 | 0.000108166 | 0.00524482 |
| GO:0048469 | cell maturation | 11/263 | 0.000108416 | 0.00524482 |
| GO:0042476 | odontogenesis | 9/263 | 0.000110349 | 0.00526989 |
| GO:0050920 | regulation of chemotaxis | 12/263 | 0.000111771 | 0.00527023 |
| GO:0050727 | regulation of inflammatory response | 17/263 | 0.00012044 | 0.00560798 |
| GO:0001836 | release of cytochrome c from mitochondria | 6/263 | 0.000122673 | 0.00564146 |
| GO:0002474 | antigen processing and presentation of peptide antigen via MHC class I | 5/263 | 0.000125031 | 0.00567976 |
| GO:0097254 | renal tubular secretion | 4/263 | 0.000128265 | 0.00575648 |
| GO:0030098 | lymphocyte differentiation | 17/263 | 0.000138577 | 0.00614524 |
| GO:0030099 | myeloid cell differentiation | 17/263 | 0.000142479 | 0.00624393 |
| GO:0043434 | response to peptide hormone | 17/263 | 0.000146477 | 0.00634448 |
| GO:0001706 | endoderm formation | 6/263 | 0.000165324 | 0.00707852 |
| GO:0001938 | positive regulation of endothelial cell proliferation | 8/263 | 0.000181146 | 0.00766784 |
| GO:0032102 | negative regulation of response to external stimulus | 18/263 | 0.000184755 | 0.00773271 |
| GO:0071384 | cellular response to corticosteroid stimulus | 6/263 | 0.000199726 | 0.00826643 |
| GO:0030198 | extracellular matrix organization | 14/263 | 0.000203853 | 0.00834453 |
| GO:0043062 | extracellular structure organization | 14/263 | 0.000210472 | 0.00852184 |
| GO:0045229 | external encapsulating structure organization | 14/263 | 0.000217276 | 0.00870272 |
| GO:0045165 | cell fate commitment | 13/263 | 0.000229876 | 0.00908434 |
| GO:0045930 | negative regulation of mitotic cell cycle | 12/263 | 0.000234044 | 0.00908434 |
| GO:0019885 | antigen processing and presentation of endogenous peptide antigen via MHC class I | 4/263 | 0.00023412 | 0.00908434 |
| GO:0002064 | epithelial cell development | 11/263 | 0.000244595 | 0.00939296 |
| GO:0007178 | transmembrane receptor protein serine/threonine kinase signaling pathway | 16/263 | 0.0002512 | 0.00954817 |
| GO:0021700 | developmental maturation | 14/263 | 0.000262215 | 0.00986617 |
| GO:0031960 | response to corticosteroid | 9/263 | 0.000275516 | 0.01026295 |
| GO:0001885 | endothelial cell development | 6/263 | 0.000285343 | 0.01052378 |
| GO:0016525 | negative regulation of angiogenesis | 9/263 | 0.000289545 | 0.0105345 |
| GO:0016055 | Wnt signaling pathway | 17/263 | 0.00029129 | 0.0105345 |
| GO:0198738 | cell-cell signaling by wnt | 17/263 | 0.000306359 | 0.01091787 |
| GO:0072089 | stem cell proliferation | 8/263 | 0.000309153 | 0.01091787 |
| GO:1905330 | regulation of morphogenesis of an epithelium | 6/263 | 0.000310683 | 0.01091787 |
| GO:2000181 | negative regulation of blood vessel morphogenesis | 9/263 | 0.000319383 | 0.01111871 |
| GO:0140747 | regulation of ncRNA transcription | 7/263 | 0.000329276 | 0.01124534 |
| GO:0002475 | antigen processing and presentation via MHC class Ib | 4/263 | 0.000332611 | 0.01124534 |
| GO:0002483 | antigen processing and presentation of endogenous peptide antigen | 4/263 | 0.000332611 | 0.01124534 |
| GO:1901343 | negative regulation of vasculature development | 9/263 | 0.000335229 | 0.01124534 |
| GO:1904037 | positive regulation of epithelial cell apoptotic process | 5/263 | 0.000338115 | 0.01124534 |
| GO:0071695 | anatomical structure maturation | 12/263 | 0.000346989 | 0.01143834 |
| GO:0033555 | multicellular organismal response to stress | 7/263 | 0.000351847 | 0.01149677 |
| GO:0010811 | positive regulation of cell-substrate adhesion | 8/263 | 0.000365414 | 0.01183625 |
| GO:0048762 | mesenchymal cell differentiation | 12/263 | 0.000371828 | 0.01194016 |
| GO:0030318 | melanocyte differentiation | 4/263 | 0.000391591 | 0.01236167 |
| GO:0035994 | response to muscle stretch | 4/263 | 0.000391591 | 0.01236167 |
| GO:0048002 | antigen processing and presentation of peptide antigen | 6/263 | 0.000397418 | 0.01244019 |
| GO:0043010 | camera-type eye development | 14/263 | 0.000410927 | 0.01269609 |
| GO:0002697 | regulation of immune effector process | 15/263 | 0.000418825 | 0.01269609 |
| GO:0010887 | negative regulation of cholesterol storage | 3/263 | 0.000424409 | 0.01269609 |
| GO:0042045 | epithelial fluid transport | 3/263 | 0.000424409 | 0.01269609 |
| GO:0003007 | heart morphogenesis | 12/263 | 0.000426043 | 0.01269609 |
| GO:0051384 | response to glucocorticoid | 8/263 | 0.000453128 | 0.01339606 |
| GO:1902893 | regulation of miRNA transcription | 6/263 | 0.000465012 | 0.01353692 |
| GO:0085029 | extracellular matrix assembly | 5/263 | 0.000465161 | 0.01353692 |
| GO:0001704 | formation of primary germ layer | 8/263 | 0.000477532 | 0.0137892 |
| GO:0061614 | miRNA transcription | 6/263 | 0.000502003 | 0.01438431 |
| GO:0071375 | cellular response to peptide hormone stimulus | 13/263 | 0.000511957 | 0.0145236 |
| GO:0046620 | regulation of organ growth | 7/263 | 0.000514662 | 0.0145236 |
| GO:0010595 | positive regulation of endothelial cell migration | 8/263 | 0.000529539 | 0.01475203 |
| GO:0043281 | regulation of cysteine-type endopeptidase activity involved in apoptotic process | 9/263 | 0.00053254 | 0.01475203 |
| GO:0043086 | negative regulation of catalytic activity | 17/263 | 0.000534637 | 0.01475203 |
| GO:0071901 | negative regulation of protein serine/threonine kinase activity | 7/263 | 0.000546834 | 0.01497761 |
| GO:0060070 | canonical Wnt signaling pathway | 13/263 | 0.000560138 | 0.01523002 |
| GO:0030595 | leukocyte chemotaxis | 11/263 | 0.000580698 | 0.01567462 |
| GO:0003156 | regulation of animal organ formation | 4/263 | 0.000613062 | 0.01642919 |
| GO:0035987 | endodermal cell differentiation | 5/263 | 0.000625183 | 0.01651637 |
| GO:0061028 | establishment of endothelial barrier | 5/263 | 0.000625183 | 0.01651637 |
| GO:0097529 | myeloid leukocyte migration | 11/263 | 0.000644566 | 0.01690851 |
| GO:0035924 | cellular response to vascular endothelial growth factor stimulus | 6/263 | 0.000673407 | 0.01752083 |
| GO:0032873 | negative regulation of stress-activated MAPK cascade | 5/263 | 0.000686723 | 0.01752083 |
| GO:0070303 | negative regulation of stress-activated protein kinase signaling cascade | 5/263 | 0.000686723 | 0.01752083 |
| GO:0071385 | cellular response to glucocorticoid stimulus | 5/263 | 0.000686723 | 0.01752083 |
| GO:0097066 | response to thyroid hormone | 4/263 | 0.00070331 | 0.01775426 |
| GO:0051090 | regulation of DNA-binding transcription factor activity | 15/263 | 0.000705404 | 0.01775426 |
| GO:0021781 | glial cell fate commitment | 3/263 | 0.000720378 | 0.0177709 |
| GO:0035810 | positive regulation of urine volume | 3/263 | 0.000720378 | 0.0177709 |
| GO:0070294 | renal sodium ion absorption | 3/263 | 0.000720378 | 0.0177709 |
| GO:0002819 | regulation of adaptive immune response | 10/263 | 0.000726823 | 0.01781194 |
| GO:0001974 | blood vessel remodeling | 5/263 | 0.000752682 | 0.01828802 |
| GO:0002237 | response to molecule of bacterial origin | 14/263 | 0.000756069 | 0.01828802 |
| GO:0048568 | embryonic organ development | 16/263 | 0.000800554 | 0.01904747 |
| GO:0051345 | positive regulation of hydrolase activity | 17/263 | 0.000803798 | 0.01904747 |
| GO:0060485 | mesenchyme development | 13/263 | 0.000815702 | 0.01904747 |
| GO:0060688 | regulation of morphogenesis of a branching structure | 5/263 | 0.00082326 | 0.01904747 |
| GO:1902895 | positive regulation of miRNA transcription | 5/263 | 0.00082326 | 0.01904747 |
| GO:1905562 | regulation of vascular endothelial cell proliferation | 5/263 | 0.00082326 | 0.01904747 |
| GO:0051896 | regulation of phosphatidylinositol 3-kinase/protein kinase B signal transduction | 11/263 | 0.000844072 | 0.01940844 |
| GO:0051347 | positive regulation of transferase activity | 15/263 | 0.000859698 | 0.01947808 |
| GO:0008360 | regulation of cell shape | 8/263 | 0.000862787 | 0.01947808 |
| GO:0071902 | positive regulation of protein serine/threonine kinase activity | 8/263 | 0.000862787 | 0.01947808 |
| GO:0010827 | regulation of glucose transmembrane transport | 6/263 | 0.000886978 | 0.01990356 |
| GO:0010745 | negative regulation of macrophage derived foam cell differentiation | 3/263 | 0.000907292 | 0.01991687 |
| GO:0033700 | phospholipid efflux | 3/263 | 0.000907292 | 0.01991687 |
| GO:0034616 | response to laminar fluid shear stress | 3/263 | 0.000907292 | 0.01991687 |
| GO:0019883 | antigen processing and presentation of endogenous antigen | 4/263 | 0.000911311 | 0.01991687 |
| GO:0008637 | apoptotic mitochondrial changes | 7/263 | 0.000914305 | 0.01991687 |
| GO:0042692 | muscle cell differentiation | 15/263 | 0.000947011 | 0.02050939 |
| GO:0014812 | muscle cell migration | 7/263 | 0.000964879 | 0.02077557 |
| GO:0060348 | bone development | 10/263 | 0.000972679 | 0.02082315 |
| GO:0045936 | negative regulation of phosphate metabolic process | 14/263 | 0.000980077 | 0.02086163 |
| GO:0003151 | outflow tract morphogenesis | 6/263 | 0.001011609 | 0.02128952 |
| GO:0034599 | cellular response to oxidative stress | 11/263 | 0.001025212 | 0.02131587 |
| GO:0097305 | response to alcohol | 11/263 | 0.001025212 | 0.02131587 |
| GO:0010458 | exit from mitosis | 4/263 | 0.001030029 | 0.02131587 |
| GO:0001666 | response to hypoxia | 12/263 | 0.001118774 | 0.02284478 |
| GO:0050679 | positive regulation of epithelial cell proliferation | 10/263 | 0.001119042 | 0.02284478 |
| GO:0007492 | endoderm development | 6/263 | 0.001149275 | 0.02314081 |
| GO:0019935 | cyclic-nucleotide-mediated signaling | 6/263 | 0.001149275 | 0.02314081 |
| GO:0071456 | cellular response to hypoxia | 8/263 | 0.001183222 | 0.02369626 |
| GO:0001659 | temperature homeostasis | 9/263 | 0.001212975 | 0.02416219 |
| GO:2000628 | regulation of miRNA metabolic process | 6/263 | 0.00122327 | 0.02416267 |
| GO:0070482 | response to oxygen levels | 13/263 | 0.001225972 | 0.02416267 |
| GO:0002062 | chondrocyte differentiation | 7/263 | 0.001251719 | 0.02442689 |
| GO:0048771 | tissue remodeling | 9/263 | 0.00125995 | 0.02444434 |
| GO:0034405 | response to fluid shear stress | 4/263 | 0.001299304 | 0.02494798 |
| GO:0071398 | cellular response to fatty acid | 4/263 | 0.001299304 | 0.02494798 |
| GO:0032496 | response to lipopolysaccharide | 13/263 | 0.001325854 | 0.02532722 |
| GO:0120161 | regulation of cold-induced thermogenesis | 8/263 | 0.001346732 | 0.0253442 |
| GO:0002476 | antigen processing and presentation of endogenous peptide antigen via MHC class Ib | 3/263 | 0.00136693 | 0.0253442 |
| GO:0045785 | positive regulation of cell adhesion | 16/263 | 0.001367566 | 0.0253442 |
| GO:2000243 | positive regulation of reproductive process | 6/263 | 0.001382146 | 0.02542639 |
| GO:0001654 | eye development | 14/263 | 0.001385653 | 0.02542639 |
| GO:0001890 | placenta development | 8/263 | 0.001405057 | 0.0254919 |
| GO:0106106 | cold-induced thermogenesis | 8/263 | 0.001405057 | 0.0254919 |
| GO:0032872 | regulation of stress-activated MAPK cascade | 9/263 | 0.001409753 | 0.0254919 |
| GO:0050767 | regulation of neurogenesis | 14/263 | 0.001419325 | 0.025541 |
| GO:0050931 | pigment cell differentiation | 4/263 | 0.001450823 | 0.02585797 |
| GO:0070633 | transepithelial transport | 4/263 | 0.001450823 | 0.02585797 |
| GO:0001755 | neural crest cell migration | 5/263 | 0.001463683 | 0.02596295 |
| GO:0001701 | in utero embryonic development | 14/263 | 0.001488725 | 0.02628199 |
| GO:0150063 | visual system development | 14/263 | 0.001524474 | 0.02647134 |
| GO:0010821 | regulation of mitochondrion organization | 8/263 | 0.001527725 | 0.02647134 |
| GO:0034614 | cellular response to reactive oxygen species | 8/263 | 0.001527725 | 0.02647134 |
| GO:0002699 | positive regulation of immune effector process | 11/263 | 0.001529205 | 0.02647134 |
| GO:0001892 | embryonic placenta development | 6/263 | 0.001556302 | 0.02647134 |
| GO:0043406 | positive regulation of MAP kinase activity | 6/263 | 0.001556302 | 0.02647134 |
| GO:0048145 | regulation of fibroblast proliferation | 6/263 | 0.001556302 | 0.02647134 |
| GO:0048864 | stem cell development | 6/263 | 0.001556302 | 0.02647134 |
| GO:0002685 | regulation of leukocyte migration | 10/263 | 0.001565044 | 0.02649905 |
| GO:0070302 | regulation of stress-activated protein kinase signaling cascade | 9/263 | 0.001573579 | 0.02652299 |
| GO:0072009 | nephron epithelium development | 7/263 | 0.00160139 | 0.02664552 |
| GO:0010092 | specification of animal organ identity | 4/263 | 0.001614235 | 0.02664552 |
| GO:0071425 | hematopoietic stem cell proliferation | 4/263 | 0.001614235 | 0.02664552 |
| GO:0071711 | basement membrane organization | 4/263 | 0.001614235 | 0.02664552 |
| GO:0007179 | transforming growth factor beta receptor signaling pathway | 10/263 | 0.001616614 | 0.02664552 |
| GO:0097067 | cellular response to thyroid hormone stimulus | 3/263 | 0.001642587 | 0.02674408 |
| GO:0001503 | ossification | 15/263 | 0.001644132 | 0.02674408 |
| GO:0048732 | gland development | 15/263 | 0.001680647 | 0.0271779 |
| GO:0090497 | mesenchymal cell migration | 5/263 | 0.001699987 | 0.0271779 |
| GO:1900024 | regulation of substrate adhesion-dependent cell spreading | 5/263 | 0.001699987 | 0.0271779 |
| GO:1905517 | macrophage migration | 5/263 | 0.001699987 | 0.0271779 |
| GO:0051403 | stress-activated MAPK cascade | 10/263 | 0.00172389 | 0.02744227 |
| GO:0007162 | negative regulation of cell adhesion | 12/263 | 0.001743165 | 0.02745294 |
| GO:0052547 | regulation of peptidase activity | 12/263 | 0.001743165 | 0.02745294 |
| GO:0048880 | sensory system development | 14/263 | 0.001754352 | 0.02745782 |
| GO:0051385 | response to mineralocorticoid | 4/263 | 0.001790013 | 0.02778497 |
| GO:0036293 | response to decreased oxygen levels | 12/263 | 0.001790172 | 0.02778497 |
| GO:2000630 | positive regulation of miRNA metabolic process | 5/263 | 0.001828096 | 0.02825585 |
| GO:0045824 | negative regulation of innate immune response | 6/263 | 0.001848232 | 0.02844903 |
| GO:0036294 | cellular response to decreased oxygen levels | 8/263 | 0.001871767 | 0.02869272 |
| GO:2000116 | regulation of cysteine-type endopeptidase activity | 9/263 | 0.001880327 | 0.02869493 |
| GO:0062197 | cellular response to chemical stress | 12/263 | 0.001887318 | 0.02869493 |
| GO:0002693 | positive regulation of cellular extravasation | 3/263 | 0.001950625 | 0.02941731 |
| GO:0042481 | regulation of odontogenesis | 3/263 | 0.001950625 | 0.02941731 |
| GO:0040036 | regulation of fibroblast growth factor receptor signaling pathway | 4/263 | 0.001978632 | 0.02971938 |
| GO:0050886 | endocrine process | 6/263 | 0.002064713 | 0.03088778 |
| GO:0045786 | negative regulation of cell cycle | 14/263 | 0.002105094 | 0.03125182 |
| GO:0051147 | regulation of muscle cell differentiation | 8/263 | 0.002105827 | 0.03125182 |
| GO:0007160 | cell-matrix adhesion | 10/263 | 0.002145661 | 0.03164419 |
| GO:1902105 | regulation of leukocyte differentiation | 12/263 | 0.002149256 | 0.03164419 |
| GO:0001916 | positive regulation of T cell mediated cytotoxicity | 4/263 | 0.00218056 | 0.03185329 |
| GO:0071548 | response to dexamethasone | 4/263 | 0.00218056 | 0.03185329 |
| GO:0031098 | stress-activated protein kinase signaling cascade | 10/263 | 0.002212168 | 0.03218877 |
| GO:0043524 | negative regulation of neuron apoptotic process | 8/263 | 0.002274246 | 0.03296329 |
| GO:0010885 | regulation of cholesterol storage | 3/263 | 0.002292317 | 0.03307658 |
| GO:0014033 | neural crest cell differentiation | 6/263 | 0.002299821 | 0.03307658 |
| GO:0045860 | positive regulation of protein kinase activity | 11/263 | 0.002346249 | 0.03361453 |
| GO:0002042 | cell migration involved in sprouting angiogenesis | 6/263 | 0.00242467 | 0.03447289 |
| GO:0014909 | smooth muscle cell migration | 6/263 | 0.00242467 | 0.03447289 |
| GO:0051091 | positive regulation of DNA-binding transcription factor activity | 10/263 | 0.00249499 | 0.03533778 |
| GO:0061351 | neural precursor cell proliferation | 8/263 | 0.002546511 | 0.03593088 |
| GO:0015909 | long-chain fatty acid transport | 5/263 | 0.002576964 | 0.03595203 |
| GO:0048645 | animal organ formation | 5/263 | 0.002576964 | 0.03595203 |
| GO:0070301 | cellular response to hydrogen peroxide | 5/263 | 0.002576964 | 0.03595203 |
| GO:0010721 | negative regulation of cell development | 11/263 | 0.002615396 | 0.0361469 |
| GO:0035136 | forelimb morphogenesis | 4/263 | 0.002626198 | 0.0361469 |
| GO:0048246 | macrophage chemotaxis | 4/263 | 0.002626198 | 0.0361469 |
| GO:0048638 | regulation of developmental growth | 12/263 | 0.002629748 | 0.0361469 |
| GO:0071397 | cellular response to cholesterol | 3/263 | 0.002668863 | 0.03654969 |
| GO:0048545 | response to steroid hormone | 12/263 | 0.002695516 | 0.03677948 |
| GO:0033002 | muscle cell proliferation | 10/263 | 0.002725581 | 0.03705398 |
| GO:1990845 | adaptive thermogenesis | 8/263 | 0.002741749 | 0.03713824 |
| GO:1901653 | cellular response to peptide | 13/263 | 0.002752452 | 0.03714812 |
| GO:0022604 | regulation of cell morphogenesis | 10/263 | 0.002806128 | 0.03769951 |
| GO:2000241 | regulation of reproductive process | 9/263 | 0.002813547 | 0.03769951 |
| GO:0052548 | regulation of endopeptidase activity | 11/263 | 0.00283321 | 0.03782691 |
| GO:0042060 | wound healing | 14/263 | 0.002919499 | 0.03883977 |
| GO:0048660 | regulation of smooth muscle cell proliferation | 8/263 | 0.00294849 | 0.03902893 |
| GO:0070371 | ERK1 and ERK2 cascade | 12/263 | 0.002972118 | 0.03902893 |
| GO:0045444 | fat cell differentiation | 10/263 | 0.002972931 | 0.03902893 |
| GO:2000177 | regulation of neural precursor cell proliferation | 6/263 | 0.002975629 | 0.03902893 |
| GO:0035809 | regulation of urine volume | 3/263 | 0.003081391 | 0.03999366 |
| GO:2000738 | positive regulation of stem cell differentiation | 3/263 | 0.003081391 | 0.03999366 |
| GO:0120162 | positive regulation of cold-induced thermogenesis | 6/263 | 0.003126895 | 0.04044334 |
| GO:0033674 | positive regulation of kinase activity | 12/263 | 0.003194337 | 0.04117268 |
| GO:0002702 | positive regulation of production of molecular mediator of immune response | 7/263 | 0.003249375 | 0.04145178 |
| GO:0034219 | carbohydrate transmembrane transport | 7/263 | 0.003249375 | 0.04145178 |
| GO:0098781 | ncRNA transcription | 7/263 | 0.003249375 | 0.04145178 |
| GO:0045619 | regulation of lymphocyte differentiation | 9/263 | 0.003296709 | 0.04191209 |
| GO:0014823 | response to activity | 5/263 | 0.00331928 | 0.04205551 |
| GO:0046661 | male sex differentiation | 8/263 | 0.0033983 | 0.04257422 |
| GO:0071453 | cellular response to oxygen levels | 8/263 | 0.0033983 | 0.04257422 |
| GO:0071526 | semaphorin-plexin signaling pathway | 4/263 | 0.003405937 | 0.04257422 |
| GO:0007265 | Ras protein signal transduction | 12/263 | 0.003429832 | 0.04272951 |
| GO:0010586 | miRNA metabolic process | 6/263 | 0.003446556 | 0.04279474 |
| GO:0010888 | negative regulation of lipid storage | 3/263 | 0.00353096 | 0.0436971 |
| GO:0009410 | response to xenobiotic stimulus | 14/263 | 0.003594376 | 0.04420549 |
| GO:0031349 | positive regulation of defense response | 15/263 | 0.003595776 | 0.04420549 |
| GO:0061326 | renal tubule development | 6/263 | 0.00361521 | 0.04429821 |
| GO:0010634 | positive regulation of epithelial cell migration | 8/263 | 0.003642295 | 0.04448376 |
| GO:0048713 | regulation of oligodendrocyte differentiation | 4/263 | 0.003697308 | 0.04500808 |
| GO:0035914 | skeletal muscle cell differentiation | 5/263 | 0.00374305 | 0.04541649 |
| GO:0043409 | negative regulation of MAPK cascade | 8/263 | 0.00376927 | 0.04558614 |
| GO:0006959 | humoral immune response | 10/263 | 0.003824149 | 0.04610018 |
| GO:0110020 | regulation of actomyosin structure organization | 6/263 | 0.003970814 | 0.0475211 |
| GO:0035272 | exocrine system development | 4/263 | 0.004005131 | 0.0475211 |
| GO:0048483 | autonomic nervous system development | 4/263 | 0.004005131 | 0.0475211 |
| GO:0001759 | organ induction | 3/263 | 0.004018563 | 0.0475211 |
| GO:0036303 | lymph vessel morphogenesis | 3/263 | 0.004018563 | 0.0475211 |
| GO:0006469 | negative regulation of protein kinase activity | 8/263 | 0.004033474 | 0.04754649 |
| GO:0051145 | smooth muscle cell differentiation | 5/263 | 0.004204251 | 0.04940327 |

**Suppl. Table S6. List of 100 upregulated genes in ESD-treated HUVECs**

| **ID** | **Gene name** | **logFC** | **p-value** | **FDR** |
| --- | --- | --- | --- | --- |
| ENSG00000187513 | **GJA4** | 1.644633 | 7.87E-05 | 0.00694605 |
| ENSG00000163209 | **SPRR3** | 1.505568 | 0.000456 | 0.020192135 |
| ENSG00000164451 | **CALHM4** | 1.29899 | 0.001213 | 0.036375888 |
| ENSG00000137033 | **IL33** | 1.231244 | 0.000237 | 0.013462414 |
| ENSG00000240583 | **AQP1** | 1.18811 | 2.12E-09 | 6.47E-06 |
| ENSG00000181444 | **ZNF467** | 1.037638 | 0.000239 | 0.013476413 |
| ENSG00000205420 | **KRT6A** | 1.008113 | 0.000138 | 0.009629272 |
| ENSG00000164116 | **GUCY1A1** | 0.994231 | 6.15E-08 | 6.25E-05 |
| ENSG00000253177 | **AC104211.2** | 0.984441 | 0.000353 | 0.017408634 |
| ENSG00000137573 | **SULF1** | 0.982443 | 0.001158 | 0.035745629 |
| ENSG00000131386 | **GALNT15** | 0.976015 | 1.49E-07 | 0.000133688 |
| ENSG00000210077 | **MT-TV** | 0.962567 | 0.000864 | 0.030545239 |
| ENSG00000179388 | **EGR3** | 0.960262 | 0.00134 | 0.038408191 |
| ENSG00000130600 | **H19** | 0.957099 | 0.001416 | 0.0397801 |
| ENSG00000247595 | **SPTY2D1OS** | 0.956231 | 0.000651 | 0.025460259 |
| ENSG00000197142 | **ACSL5** | 0.946992 | 0.00075 | 0.028170517 |
| ENSG00000072071 | **ADGRL1** | 0.946165 | 4.04E-05 | 0.004586609 |
| ENSG00000172349 | **IL16** | 0.945603 | 0.00017 | 0.010705181 |
| ENSG00000247157 | **LINC01252** | 0.941731 | 0.001191 | 0.036190606 |
| ENSG00000102760 | **RGCC** | 0.939808 | 0.001685 | 0.042960824 |
| ENSG00000111341 | **MGP** | 0.926522 | 2.31E-12 | 3.52E-08 |
| ENSG00000156298 | **TSPAN7** | 0.915498 | 0.001994 | 0.047288666 |
| ENSG00000196460 | **RFX8** | 0.903525 | 0.001427 | 0.03999793 |
| ENSG00000004799 | **PDK4** | 0.898393 | 3.15E-05 | 0.004031251 |
| ENSG00000107731 | **UNC5B** | 0.897576 | 5.73E-09 | 1.41E-05 |
| ENSG00000123358 | **NR4A1** | 0.895825 | 0.000166 | 0.01052693 |
| ENSG00000203883 | **SOX18** | 0.895482 | 0.000622 | 0.024615185 |
| ENSG00000170345 | **FOS** | 0.884413 | 5.19E-05 | 0.005251874 |
| ENSG00000129757 | **CDKN1C** | 0.879611 | 3.06E-08 | 4.67E-05 |
| ENSG00000164849 | **GPR146** | 0.873616 | 5.98E-06 | 0.001435921 |
| ENSG00000163624 | **CDS1** | 0.872799 | 8.96E-05 | 0.007426193 |
| ENSG00000179855 | **GIPC3** | 0.852643 | 1.32E-05 | 0.002340831 |
| ENSG00000105270 | **CLIP3** | 0.84676 | 1.28E-05 | 0.002304787 |
| ENSG00000065717 | **TLE2** | 0.83976 | 8.39E-05 | 0.007221011 |
| ENSG00000169418 | **NPR1** | 0.839164 | 9.58E-06 | 0.002028001 |
| ENSG00000118777 | **ABCG2** | 0.820447 | 2.70E-06 | 0.0008827 |
| ENSG00000175899 | **A2M** | 0.809227 | 1.40E-05 | 0.002353314 |
| ENSG00000125744 | **RTN2** | 0.79511 | 0.001153 | 0.035745629 |
| ENSG00000209082 | **MT-TL1** | 0.793989 | 1.13E-05 | 0.002169599 |
| ENSG00000167984 | **NLRC3** | 0.791033 | 0.001972 | 0.047094856 |
| ENSG00000153234 | **NR4A2** | 0.787119 | 3.02E-05 | 0.003899879 |
| ENSG00000244414 | **CFHR1** | 0.772471 | 0.000988 | 0.032966682 |
| ENSG00000164692 | **COL1A2** | 0.767171 | 5.57E-08 | 6.25E-05 |
| ENSG00000132170 | **PPARG** | 0.758282 | 0.00152 | 0.040805511 |
| ENSG00000112183 | **RBM24** | 0.750486 | 0.000409 | 0.019209042 |
| ENSG00000136826 | **KLF4** | 0.742623 | 0.000238 | 0.013476413 |
| ENSG00000159640 | **ACE** | 0.740375 | 1.87E-09 | 6.47E-06 |
| ENSG00000182492 | **BGN** | 0.736283 | 4.45E-06 | 0.001191105 |
| ENSG00000091972 | **CD200** | 0.733854 | 2.67E-05 | 0.003609394 |
| ENSG00000121966 | **CXCR4** | 0.73125 | 9.14E-10 | 6.47E-06 |
| ENSG00000168758 | **SEMA4C** | 0.711354 | 0.001643 | 0.042323402 |
| ENSG00000145703 | **IQGAP2** | 0.70526 | 0.000287 | 0.015359873 |
| ENSG00000057657 | **PRDM1** | 0.687412 | 0.000409 | 0.019209042 |
| ENSG00000188191 | **PRKAR1B** | 0.681357 | 0.001607 | 0.041943013 |
| ENSG00000247134 | **AC090204.1** | 0.678509 | 0.000828 | 0.029896979 |
| ENSG00000174059 | **CD34** | 0.666445 | 4.43E-08 | 6.14E-05 |
| ENSG00000213203 | **GIMAP1** | 0.662826 | 6.11E-08 | 6.25E-05 |
| ENSG00000245060 | **LINC00847** | 0.658058 | 3.47E-05 | 0.004138814 |
| ENSG00000139597 | **N4BP2L1** | 0.656202 | 0.000215 | 0.012549949 |
| ENSG00000130208 | **APOC1** | 0.64981 | 0.001208 | 0.036375888 |
| ENSG00000210112 | **MT-TM** | 0.641747 | 0.001254 | 0.037126249 |
| ENSG00000064042 | **LIMCH1** | 0.624703 | 1.45E-09 | 6.47E-06 |
| ENSG00000196605 | **ZNF846** | 0.61801 | 0.001949 | 0.047094856 |
| ENSG00000104081 | **BMF** | 0.609743 | 0.000141 | 0.009701796 |
| ENSG00000185305 | **ARL15** | 0.609187 | 1.23E-06 | 0.000533932 |
| ENSG00000164056 | **SPRY1** | 0.607243 | 2.52E-05 | 0.003558253 |
| ENSG00000171223 | **JUNB** | 0.603709 | 4.42E-05 | 0.004810685 |
| ENSG00000179163 | **FUCA1** | 0.602316 | 0.000217 | 0.012594541 |
| ENSG00000184557 | **SOCS3** | 0.601762 | 0.001371 | 0.039068103 |
| ENSG00000163132 | **MSX1** | 0.600013 | 7.52E-06 | 0.001692722 |
| ENSG00000135363 | **LMO2** | 0.59857 | 2.78E-05 | 0.003669648 |
| ENSG00000020181 | **ADGRA2** | 0.591152 | 1.49E-05 | 0.002420814 |
| ENSG00000165507 | **DEPP1** | 0.589237 | 5.60E-08 | 6.25E-05 |
| ENSG00000170323 | **FABP4** | 0.587604 | 5.21E-06 | 0.001302021 |
| ENSG00000101445 | **PPP1R16B** | 0.584268 | 2.68E-05 | 0.003609394 |
| ENSG00000143341 | **HMCN1** | 0.583936 | 0.001678 | 0.042869306 |
| ENSG00000037280 | **FLT4** | 0.577957 | 8.93E-06 | 0.001945066 |
| ENSG00000239917 | **RPS10P16** | 0.577165 | 0.001928 | 0.046800115 |
| ENSG00000141295 | **SCRN2** | 0.572021 | 0.00016 | 0.01032774 |
| ENSG00000079257 | **LXN** | 0.568081 | 0.0001 | 0.007803597 |
| ENSG00000095713 | **CRTAC1** | 0.567726 | 0.001735 | 0.043798338 |
| ENSG00000136160 | **EDNRB** | 0.567582 | 0.000304 | 0.015973332 |
| ENSG00000072163 | **LIMS2** | 0.563017 | 1.48E-05 | 0.002420814 |
| ENSG00000244694 | **PTCHD4** | 0.55988 | 0.001359 | 0.038871257 |
| ENSG00000126803 | **HSPA2** | 0.559589 | 0.000601 | 0.024182108 |
| ENSG00000135929 | **CYP27A1** | 0.557997 | 0.000124 | 0.00900363 |
| ENSG00000146859 | **TMEM140** | 0.557724 | 2.84E-06 | 0.0008827 |
| ENSG00000214870 | **AC004540.1** | 0.557281 | 0.002008 | 0.047474621 |
| ENSG00000108840 | **HDAC5** | 0.555575 | 0.001712 | 0.043352093 |
| ENSG00000126705 | **AHDC1** | 0.553902 | 0.000113 | 0.008455519 |
| ENSG00000223583 | **AL513365.1** | 0.550342 | 0.002125 | 0.049444454 |
| ENSG00000171105 | **INSR** | 0.546971 | 4.06E-07 | 0.000269246 |
| ENSG00000162496 | **DHRS3** | 0.545116 | 1.39E-06 | 0.000571694 |
| ENSG00000100234 | **TIMP3** | 0.542608 | 4.69E-06 | 0.001212696 |
| ENSG00000280852 | **AC025048.6** | 0.538321 | 0.000679 | 0.026077161 |
| ENSG00000160179 | **ABCG1** | 0.537818 | 4.94E-07 | 0.000301262 |
| ENSG00000136720 | **HS6ST1** | 0.537587 | 0.000137 | 0.009629272 |
| ENSG00000137878 | **GCOM1** | 0.536873 | 0.001118 | 0.035442564 |
| ENSG00000168672 | **FAM84B** | 0.534249 | 9.03E-05 | 0.007426193 |
|  |  |  |  |  |

**Suppl. Table S7. List of pathways modulated in miR-29 +C4a treated HUVEC**

| **ID** | **Description** | **GeneRatio** | **p-value** | **p-adjusted** |
| --- | --- | --- | --- | --- |
| GO:0003341 | cilium movement | 16/332 | 1.10E-06 | 0.004039469 |
| GO:1990868 | response to chemokine | 10/332 | 1.21E-05 | 0.005531114 |
| GO:0001539 | cilium or flagellum-dependent cell motility | 13/332 | 1.13E-05 | 0.005531114 |
| GO:0120316 | sperm flagellum assembly | 7/332 | 1.38E-05 | 0.005624414 |
| GO:0030595 | leukocyte chemotaxis | 15/332 | 2.16E-05 | 0.007934074 |
| GO:0070098 | chemokine-mediated signaling pathway | 9/332 | 3.93E-05 | 0.011576601 |
| GO:0007163 | establishment or maintenance of cell polarity | 14/332 | 5.89E-05 | 0.014394445 |
| GO:0071696 | ectodermal placode development | 4/332 | 8.69E-05 | 0.017699856 |
| GO:0006935 | chemotaxis | 21/332 | 9.95E-05 | 0.018079735 |
| GO:1990266 | neutrophil migration | 10/332 | 0.000101893 | 0.018079735 |
| GO:0003351 | epithelial cilium movement involved in extracellular fluid movement | 6/332 | 0.000108528 | 0.018079735 |
| GO:0007286 | spermatid development | 13/332 | 0.000125016 | 0.019090962 |
| GO:0006858 | extracellular transport | 6/332 | 0.000159415 | 0.023370191 |
| GO:0048515 | spermatid differentiation | 13/332 | 0.000180248 | 0.025408091 |
| GO:0071222 | cellular response to lipopolysaccharide | 13/332 | 0.000196951 | 0.026734242 |
| GO:0060326 | cell chemotaxis | 16/332 | 0.00022176 | 0.02877323 |
| GO:0044458 | motile cilium assembly | 7/332 | 0.000227674 | 0.02877323 |
| GO:0043491 | phosphatidylinositol 3-kinase/protein kinase B signal transduction | 15/332 | 0.000334559 | 0.040361304 |
| GO:0071219 | cellular response to molecule of bacterial origin | 13/332 | 0.000341392 | 0.040361304 |
| GO:0097529 | myeloid leukocyte migration | 13/332 | 0.000355516 | 0.040717653 |
| GO:0007416 | synapse assembly | 12/332 | 0.000395177 | 0.043888577 |
| GO:0051345 | positive regulation of hydrolase activity | 20/332 | 0.000415034 | 0.044738175 |
| GO:0097530 | granulocyte migration | 10/332 | 0.000435575 | 0.04537905 |
| GO:0009615 | response to virus | 18/332 | 0.000445742 | 0.04537905 |
| GO:0071621 | granulocyte chemotaxis | 9/332 | 0.000465399 | 0.04609968 |
| GO:0007030 | Golgi organization | 10/332 | 0.000507682 | 0.047858165 |
| GO:0032496 | response to lipopolysaccharide | 16/332 | 0.000509268 | 0.047858165 |

**Suppl. Table 8. List of down-regulated target gene of miR-29a-3p in endothelial cells**

| **mim-29** | **mim-29+C4** |
| --- | --- |
| DNMT3B | SPARC |
| SPARC | CD276 |
| CD276 | MYCN |
| MYCN | COL4A1 |
| BCL7A | TDG |
| COL4A1 | COL1A2 |
| TDG | COL4A2 |
| COL1A2 | FBN1 |
| COL4A2 | CCNA2 |
| FBN1 | CTNNBIP1 |
| CCNA2 | SERPINH1 |
| CTNNBIP1 | COL5A2 |
| SERPINH1 | DAG1 |
| COL5A2 | LOX |
| DAG1 | MAZ |
| LOX | SERPINB9 |
| MAZ | PPT1 |
| SERPINB9 | PXDN |
| PPT1 | TFEB |
| PXDN | FSTL1 |
| TFEB | CBX6 |
| FSTL1 | NKIRAS2 |
| CBX6 | EDC3 |
| NKIRAS2 | PIGS |
| PIGS | C1QTNF6 |
| C1QTNF6 | KLHDC3 |
| KLHDC3 | SLC2A14 |
| IGF1 | IGF1 |
| TUBB2A | TUBB2A |
| SAPCD2 | SAPCD2 |
| P3H1 | P3H1 |
| NID1 | NID1 |
| LMNB1 | LMNB1 |
| F11R | F11R |
| IGFBP3 | IGFBP3 |
| PRDM1 | PRDM1 |
| BLMH | BLMH |
| PLK1 | PLK1 |
| TTYH3 | TTYH3 |
| CCNF | CCNF |
| COL5A1 | COL5A1 |
| EFNB2 | LPAR1 |
| HSD17B2 | EFNB2 |
| NDST1 | HSD17B2 |
| ISG20 | NDST1 |
| KIF11 | ISG20 |
| LOXL2 | KIF11 |
| MKI67 | LOXL2 |
| MMP14 | MMP14 |
| MYBL2 | MYBL2 |
| PDK4 | PDK4 |
| PPIC | PPIC |
| PTX3 | PTX3 |
| RRM2 | RRM2 |
| SMS | SMS |
| TOP2A | TOP2A |
| KIF20A | AIMP2 |
| SPRY1 | KIF20A |
| VASH1 | PIM2 |
| BTBD3 | VASH1 |
| CHIC2 | SDF2L1 |
| EHD3 | CHIC2 |
| EHD2 | EHD3 |
| SPDL1 | EHD2 |
| ASF1B | ASF1B |
| ENTPD7 | ZNF512B |
| RMND5A | RMND5A |
| GGCT | GGCT |
| ANKRD13A | SLC25A22 |
| TMEM88 | FAM136A |
| HAUS8 | TMEM88 |
| MRFAP1 | MRFAP1 |
| IRGQ | CCSAP |
| SLC16A14 | SLC16A14 |
| SLC35B2 | SLC35B2 |
| CTXN1 | CCNB2 |
| CCNB2 | PEG10 |
| PEG10 | RGS4 |
| C2orf68 | AQP1 |
| RGS4 | SLC19A1 |
| PIK3R3 | ZNF282 |
| AQP1 | CLDN11 |
| SLC19A1 | TAF11 |
| CLDN11 | NIPSNAP3A |
| TAF11 | PRMT6 |
| TPX2 | SH3BP5L |
| NIPSNAP3A | ADAM19 |
| PRMT6 | SLC43A2 |
| OSTC | XXYLT1 |
| ADAM19 | HAPLN3 |
| FAM78A | MPZL3 |
| XXYLT1 | POMK |
| DTWD2 | TRIL |
| HAPLN3 | STMP1 |
| MPZL3 | POGLUT2 |
| POMK | H2BC21 |
| TRIL | CAVIN1 |
| STMP1 | H2BC9 |
| POGLUT2 | H4C3 |
| CAVIN1 |  |
| H2BC9 |  |

| **Suppl. Table S9. Characteristics of lung cancer patients and heavy-smokers cancer-free individuals** | | | | | | | |
| --- | --- | --- | --- | --- | --- | --- | --- |
|  | **Discovery set** | | **Training set** | | | **Validation set** |  |
| Characteristics | **ESA** (n=20) | **ESD** (n=20) | **ESA** (n=15) | **ESD** (n=15) | **HS**  (n=8) | **ES**  (n=89) |  |
| **Age, years** (mean**±** SD) | 68±9.5) | 69±7.8 | 69.2±8.6 | 70.7±6.5 | 62±6.5 | 68.8±10.3 |  |
| **Sex, n (%)**  Man  Women | 13 (65%)  7 (35%) | 19 (95%)  1 (5%) | 12 (80%)  3 (20%) | 12 (80%)  3 (20%) | 6 (75%)  2 (25%) | 49 (55%)  40 (45%) |  |
| **Histology, n (%)**  ADC  SCC  Other | 14 (70%)  5 (25%)  1 (1%) | 9 (45%)  7 (35%)  4 (40%) | 8 (53%)  2 (13%)  5 (33%) | 6 (40%)  4 (27%)  5 (33%) | 0 (0)  0 (0)  0 (0) | 75 (84%)  5 (6%)  9 (10%) |  |
| **Stage, n (%)**  I  II | 10 (50%)  10 (50%) | 10 (50%)  10 (50%) | 8 (53%)  7 (47%) | 8 (53%)  7 (47%) | 0 (0)  0 (0) | 66 (74%)  23 (26%) |  |
| **Overall survival**  **Alive**  **Dead** | 20  0 | 0  20 | 15  0 | 0  15 | 0  0 | 74  15 |  |

**Suppl. Table S10. Integrin staining for FC analysis**

| **Name ab** | **Concentration/sample** | **Fluorocrome** | **Supplier** |
| --- | --- | --- | --- |
| **ITGA6 (CD49f)** | 0.2 ug/ml | SuperBright 436 | eBioscience |
| **ITGB1 (CD29)** | 0.02 ug/ml | PE-Cy7 | eBioscience |
| **ITGB4 (CD104)** | 0,5 ul | APC | Invitrogen |
| **ITGA2 (CD49b)** | 0.2 ug/ml | BV510 | BD Bioscience |
| **ITGA3 (CD49c)** | 0.1 ug/ml | FITC | Invitrogen |
| **ITGAV (CD51)** | 0.2 ug/ml | PE | Life Technologies |
| **ITGB3 (CD61)** | 0.2 ug/ml | BB700 | BD Bioscience |
| **ITGA4 (CD49d)** | 0.2 ug/ml | APC-H7 | BD Bioscience |
| **ITGB5** | 0.1 ug/ml | PE | Biolegend |

**Suppl. Table S11. Antibody list for immune profiling analysis**

| **Antibody** | **Fluorochrome** | **Supplier** |
| --- | --- | --- |
| **CD49b (DX5)** | FITC | eBioscience |
| **CXCR4** | PerCP-eFluor710 | eBioscience |
| **SIGLEC-F** | BV421 | BD |
| **CD11b** | BV510 | BD |
| **Ly-6G** | BV650 | BD |
| **F4-80** | BV785 | BioLegend |
| **Ly-6C** | APC | BioLegend |
| **CD45** | PE | BioLegend |
| **CD11c** | PE-Cy7 | eBioscience |

**SUPPLEMENTARY FIGURE LEGENDS**

**Supplementary Figure S1. Characterization of lung cancer patient’s plasma-EVPs**. **A)** Size distribution and concentration of plasma derived EVPs from HS, ESA and ESD analyzed by NTA (n=5 per group). **B)** Representative TEM images showing the spherical morphology and size distribution of EVPs**. C-D)** Analysis of conventional EV markers on HS, ESA and ESD samples showing the presence of CD9, CD63, and CD81 tetraspanins (n=5 per group) by flow cytometry (**C**) or western blot (**D**). **E**) Analysis of heterodimers presence on EVPs surface; α6-β4 linked to lung tropism; αV-β3 linked to bone tropism; α4-β1 ligand of VCAM1 (left) **F)** Profiling of plasma-EVPs markers determined by flow cytometry. Graphs show Median Fluorescence Intensity (MFI) of surface markers normalized on the MFI of CD9, CD81 and CD63. **G)** The graphs show the absolute quantification of miR-1307-3p and miR-451a in plasma derived EVPs analysed with dPCR. The results are shown as miRNAs number of copies detected in 60 μg for each group HS, ESA and ESD EVPs. **H)** Volcano plot showing the gene names of proteins found to be differentially present in ESD vs HS and in ESA vs HS-EVPs. ***p<0.0001, **p<0.001, *p<0.005. The data are expressed as mean and SEM values.

**Supplementary Figure S2. C4A localization on EVPs surface. A)** C4A quantification by ELISA after high salt washing (1M NaCl) of ESD-EVPs. Incubation in PBS was used as control (n=6). **B)** ELISA assay for C4A of EVPs from healthy donors incubated with the plasma of ESD patients depleted by their EVPs (n=4). PBS **C)** Quantification of free C4A in the plasma of HD, HS, ESA and ESD patients depleted from their EVPs (n=3 per group). Data are expressed as mean and SEM. *p<0.05.

**Supplementary Figure S3. ROC curve analysis of individual and combined biomarkers.** Receiver operating characteristic (ROC) curves showing the diagnostic performance of miR-29, C4A, and their combined index in distinguishing deceased and surviving patients in the training and validation set, respectively. The area under the curve (AUC) values, and respective 95% Confidence Interval (CI), are reported.

**Supplementary Figure S4.** **EVPs modulated the phenotype of endothelial cells** **A)** Representative images of control (PBS+CFSE) and EV-CFSE particle analysis showing gating strategy: bigger EVs (300nm-1um); microvesicles (150-300nm) and small extracellular vesicles or exosomes (50-150 nm); quantification of particle numbers is indicated in the tables below. Representative images of EVPs labelled with CFSE (left) (visualized in the different channels) **B)** Immunofluorescence staining of 3D models after 2 weeks of culture: Endothelial cells positive for vWF in green; DAPI (blue) was used for nuclear staining (4 channels, 8 frames each). **C)** ELISA quantification of CCL2 and CXCL2 released by endothelial cells after treatment with ESD or ESA-EVPs. **D)** Adhesion of human monocytes (CD14+ cells) to endothelial cells treated with ESA or ESD. The data are expressed as percentage of double positive cells PKH26+/CD14+ detected by flow cytometry. **E)** Representative images and quantification of the number of intersections formed by HUVECs on the Matrigel layer after ESA and ESD treatment. Untreated cells were used as a control. (n=3 per group). **F)** Flow Cytometry analysis of HUVEC treated with lipoprotein or small EVs (sEV) fractions of ESD-EVPs after sucrose density gradient separation. Untreated cells were used as a control. (n=4)

**Supplementary Figure S5. Effect of EVP treatment on fibroblasts.** **A)** qPCR analysis of key genes of fibroblast phenotype upon EVP treatment for 48 hours. Untreated cells were used as control. **B)** Heatmap showing the mRNA levels of several genes upon the treatment with EVPs (2 treatments of 72 hours each) of fibroblast cultured in 3D conditions. Untreated constructs were used as control **C)** CXCL1 levels in CM of CXCL1 silenced compared to SCR. **D) a-**3D rendering model of bioprinting geometry and layering. Three different PF-based bioinks were used in bioprinting. The bioinks were alternated every 4 layers from bottom to top containing CCD19lu fibroblast, HUVECs, and HBEC-KRAS, respectively; **b-** Microfluidic printing head (MPH) with a co-axial nozzle extruder during the additive manufacturing process. The red arrow indicates the triculture construct during its bioprinting; **c-** Bioprinted multi-cellular 12-layers thick construct. Scale bar represent 8 mm; **d-f-** Representative bright-field and immunofluorescence images of the bioprinted triculture on day 0. HBEC-KRAS cells were labelled with Dir+, HUVECs with PKH-67 (green), and fibroblasts with PKH-26 (red). Scale bars represent 250 µm, 200 µm, and 100 µm, respectively; **g-i-** Representative immunofluorescence images at 40X of the bioprinted triculture on day 14. Fibroblasts were labelled in red, HUVECs in green, and HBECs in magenta. Scale bars represent 100 µm. **E)** Analysis of the number of copies by dPCR of specific mRNAs on sorted cells after EVPs administration (n=1). Data are expressed as mean and SEM values. *p<0.05.

**Supplementary Figure S6. A)** mRNA relative expression of several genes in the lung of treated mice with ESA and ESD; untreated mice were used as control (n=8 for ESA and ESD; n=7 for untreated). **B)** mRNA relative expression of CXCL1, CD206, VCAM1 and IL-6 in the liver of treated mice with ESA and ESD; untreated mice were used as control (n=4 per group). **C)** The graphs show the percentage of the different subsets of immune cells (only myeloid compartment) detected by flow cytometry in the lungs of mice upon EVP treatment and expressed as fold increase. **D)** Representative images of neutrophils extracellular traps. Data are expressed as mean and SEM. *p<0.05; **p<0.01; ***p<0.001.

**Supplementary Figure S7. A)** Gating strategy for lung colonization analysis **B)** Analysis of immune cells detected in the lung of mice after 45 days from the i.v of cancer cells. The graphs show the percentage of the different subsets of immune cells (only myeloid compartment) detected by flow cytometry (ex vivo) and expressed as fold increase. Data are expressed as mean and SEM.

**Supplementary Figure S8. Endothelial gene expression after mimic transfection**. **A)** Bar chart showing the mRNA relative expression of VCAM1, CXCR4, CXCL1, CXCL2, CCL2 and SPARC on HUVEC cells treated with mim-199a and mim-29a by qPCR. SCR-miRNA treated cells were used as a control. **B)** Flow cytometry dot plot showing the presence of PAR4 receptor on endothelial cells (n=3). **C)** mRNA relative expression of SPARC after mim-29 and C4a administration. **D)** SPARC mRNA relative expression in HUVECs after silencing of SPARC and concomitant addition of C4a. Data are expressed as mean and SEM *p<0.05; **p<0.01; ***p<0.001; ****p<0.0001

1 Forsonits, A. I. *et al.* Improved Accessibility of Extracellular Vesicle Surface Molecules Upon Partial Removal of the Protein Corona by High Ionic Strength. *J Extracell Vesicles* **14**, e70124 (2025). <https://doi.org/10.1002/jev2.70124>

2 Agrawal, A. *et al.* Mechanical signatures in cancer metastasis. *NPJ Biol Phys Mech* **2**, 3 (2025). <https://doi.org/10.1038/s44341-024-00007-x>

3 Cui, C., Schoenfelt, K. Q., Becker, K. M. & Becker, L. Isolation of polymorphonuclear neutrophils and monocytes from a single sample of human peripheral blood. *STAR Protoc* **2**, 100845 (2021). <https://doi.org/10.1016/j.xpro.2021.100845>

4 O'Farrell, H. E., Bowman, R. V., Fong, K. M. & Yang, I. A. Plasma Extracellular Vesicle miRNAs Can Identify Lung Cancer, Current Smoking Status, and Stable COPD. *Int J Mol Sci* **22** (2021). <https://doi.org/10.3390/ijms22115803>

5 Conte, D. *et al.* Novel method to detect microRNAs using chip-based QuantStudio 3D digital PCR. *BMC Genomics* **16**, 849 (2015). <https://doi.org/10.1186/s12864-015-2097-9>
